# 2-(4-Fluorophenyl)-1*H*-benzo[*d*]imidazole as a Promising Template for the Development of Metabolically Robust, α1β2γ2GABA-A Receptor-Positive Allosteric Modulators

**DOI:** 10.1021/acschemneuro.2c00800

**Published:** 2023-02-27

**Authors:** Monika Marcinkowska, Nikola Fajkis-Zajączkowska, Katarzyna Szafrańska, Jakub Jończyk, Agata Siwek, Barbara Mordyl, Tadeusz Karcz, Gniewomir Latacz, Marcin Kolaczkowski

**Affiliations:** † Department of Medicinal Chemistry, Faculty of Pharmacy, Jagiellonian University Medical College, 9 Medyczna Street, 30-688 Kraków, Poland; ‡ Department of Pharmacobiology, Faculty of Pharmacy Jagiellonian University Medical College, 9 Medyczna Street, 30-688 Kraków, Poland; § Department of Technology and Biotechnology of Drugs, Faculty of Pharmacy, Jagiellonian University Medical College, 9 Medyczna Street, 30-688 Kraków, Poland

**Keywords:** 1H-benzo[d]imidazole, GABA-A receptor, positive allosteric modulator, benzodiazepine site

## Abstract

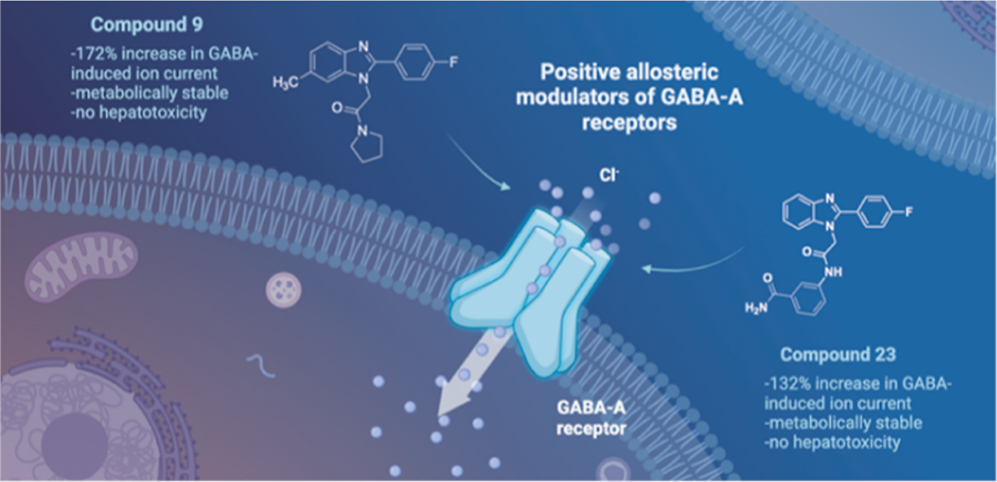

Modulation of α1β2γ2GABA-A receptor subpopulation expressed in the basal ganglia region is a conceptually novel mode of pharmacological strategy that offers prospects to tackle a variety of neurological dysfunction. Although clinical findings provided compelling evidence for the validity of this strategy, the current chemical space of molecules able to modulate the α1/γ2 interface of the GABA-A receptor is limited to imidazo[1,2-*a*]pyridine derivatives that undergo rapid biotransformation. In response to a deficiency in the chemical repertoire of GABA-A receptors, we identified a series of 2-(4-fluorophenyl)-1*H*-benzo[*d*]imidazoles as positive allosteric modulators (PAMs) with improved metabolic stability and reduced potential for hepatotoxicity, where lead molecules **9** and **23** displayed interesting features in a preliminary investigation. We further disclose that the identified scaffold shows a preference for interaction with the α1/γ2 interface of the GABA-A receptor, delivering several PAMs of the GABA-A receptor. The present work provides useful chemical templates to further explore the therapeutic potential of GABA-A receptor ligands and enriches the chemical space of molecules suitable for the interaction with the α1/γ2 interface.

## 1 Introduction

**Figure 1:**
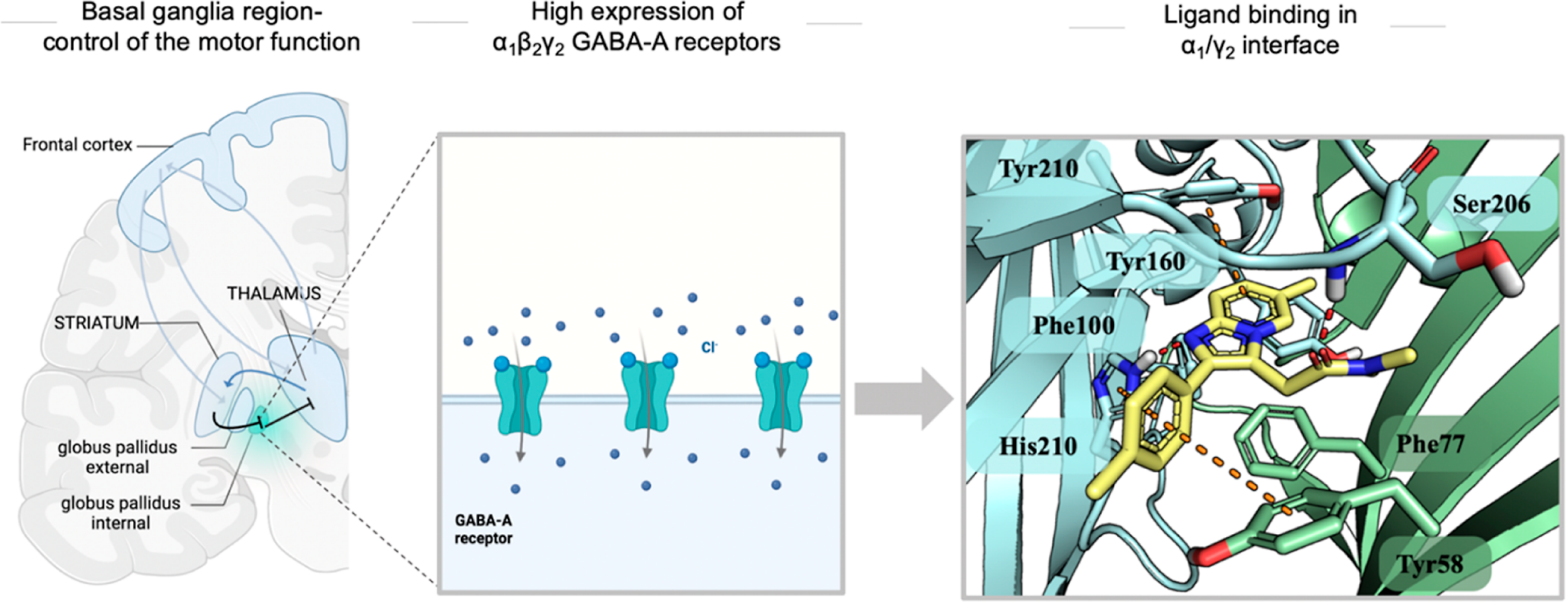
Basal ganglia region and a ligand (zolpidem) bound to the GABA-A receptor at the α1/γ2 interface.^1−3^

The introduction of GABA-A receptor modulators to clinics has revolutionized short-term insomnia therapy, making them among the top-selling drugs on the market.^1,2^ Intriguingly, recent reports refer to novel uses, beyond insomnia, such as a transient reversal of brain stroke symptoms or an improvement in motor function in subjects with Parkinson’s disease.^1,2^ The latter activities were reported solely for drug binding via the α1/γ2 interface of the α1β2γ2 GABA-A receptor subpopulation, highly expressed in the basal ganglia region, which is crucial in orchestrating motor functions (Figure 1).^3^ This feature offers prospects for the development of long-term therapeutic agents that exploit the modulation of the α1β2γ2 GABA-A receptor, against an array of neurological dysfunction related to the basal ganglia region.^1,2^

**Figure 2:**
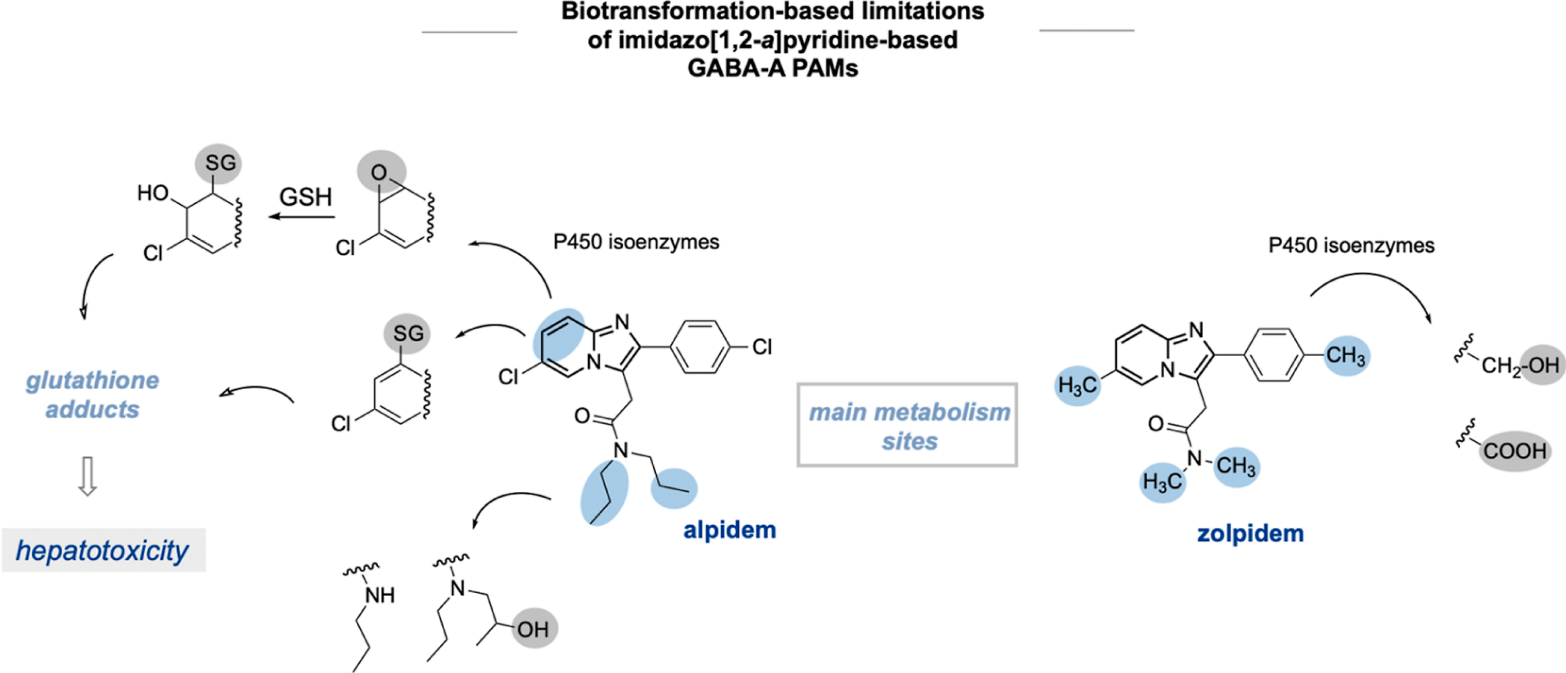
Reported positive allosteric modulators (PAMs) of the GABA-A receptor based on the imidazo[1,2-*a*]pyridine scaffold and their main metabolic sites, conferring high metabolic degradation.^7^ GSH—glutathione.

Despite the availability of a whole palette of GABA-A receptor ligands, only a few chemical modalities have been reported to target α1β2γ2 GABA-A receptor subpopulation and act via the allosteric recognition site between the α1/γ2 interface.^2,4,5^ These refer to compounds bearing mainly an imidazo[1,2-*a*]pyridine template, such as zolpidem and alpidem (Figure 1). Although these agents are excellent nanomolar binders of α1β2γ2GABA-A receptors, within the α1/γ2 interface, they are characterized by high metabolic vulnerability,^6^ which is suitable for rapid insomnia treatment, but would be challenging for long-term treatments in neurological patients. In the case of alpidem, the formation of the toxic epoxide metabolite that deprives the glutathione reservoir,^7,8^ induces hepatotoxicity,^11^ and poses an additional hurdle (Figure 2). This particular feature led to the final withdrawal of alpidem from the pharmaceutical marked.^9^ The limited chemical repertoire of α1β2γ2 GABA-A receptor ligands, which is narrowed to metabolically unstable imidazo[1,2-*a*]pyridine derivatives, has encouraged the exploration of new areas of chemical space and the validation of novel metabolically robust structural motifs.

The 2-phenyl-1*H*-benzo[*d*]imidazole is a widespread motif in a plethora of bioactive compounds, although it has been rarely exploited in the GABA-A receptor chemical repertoire.^10^ The confidence in this scaffold steams out from its stereoelectronic feature and drug-like properties owing to fit optimally in the Ro5 space. Indeed, 1*H*-benzo[*d*]imidazoles have been delineated as one of the top 100 most commonly encountered ring systems in the structure of FDA-approved drugs.^11^ The relatively low molecular weight combined with low basicity (p*K*_a_ around 5) renders 1*H*-benzo[*d*]imidazoles suitable for harnessing in the chemical structure of potential GABA-A receptor modulators. Moreover, 1*H*-benzo[*d*]imidazoles represent a bioisostere of the imidazo[1,2-*a*]pyridine motif and thus may mimic its selectivity toward the region of the α1/γ2 interface within the α1β2γ2 GABA-A receptor.

These data inspired us to explore the biological potential of benzimidazoles as α1β2γ2 GABA-A receptor-PAMs. Alongside, we performed subtle molecular editing with the aim of enhancing the metabolic stability of novel compounds. Within this context, we have identified a series of 2-(4-fluorophenyl)-1*H*-benzo[*d*]imidazoles as metabolically stabile ligands of the GABA-A receptor that act as PAMs of the GABA-A receptor. Our studies are complemented by a molecular docking study which discloses structural features essential for molecular recognition with the GABA-A receptor. Furthermore, we provide a structural basis for the development of metabolically stable scaffolds that interact with the α1β2γ2 GABA-A receptor, laying the foundation for the generation of new lead structures to confront pathologies related to the dysfunction of the basal ganglia region.

## 2 Results and Discussion

### 2.1 Design and Synthesis

**Figure 3:**
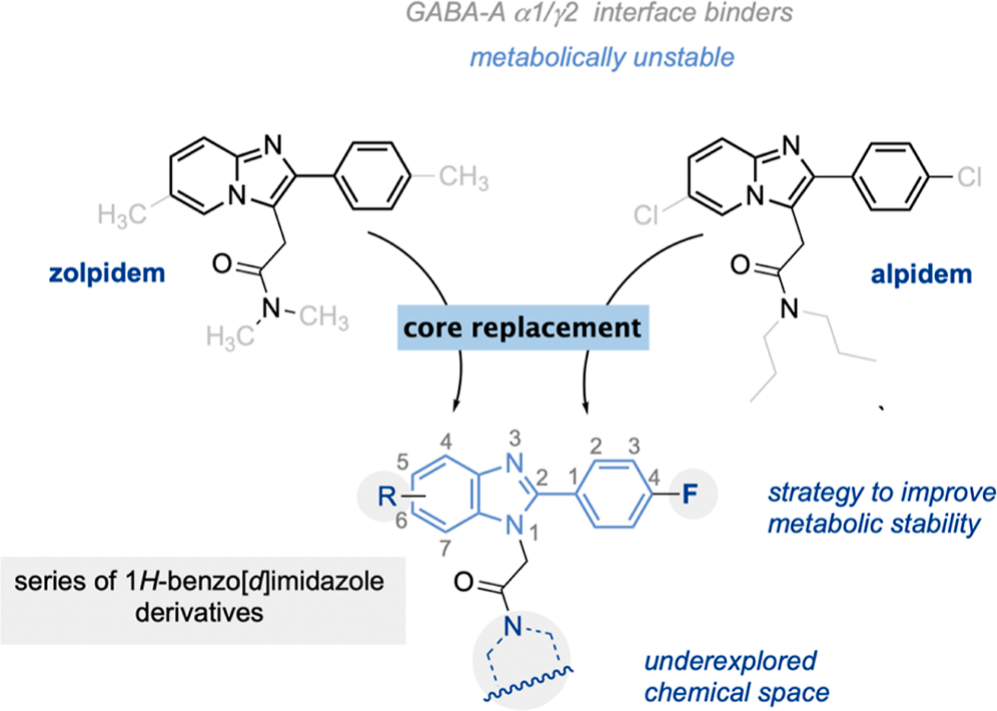
Design concept of a series of 1*H*-benzo[*d*]imidazoles toward improved metabolic stability. R = CH_3_ or H.

Relying on the structural requirements responsible for the molecular interaction with the allosteric recognition site within the α1 and γ2 interface of the GABA-A receptor, we composed a set of potential ligands bearing the 1*H*-benzo[*d*]imidazoles motif.^12−14^ We found that replacement of the 2-phenylimidazo[1,2-*a*]pyridine core presented in the structure of zolpidem and alpidem with the 2-phenyl-1*H*-benzo[*d*]imidazole template showed an overlap of the core pharmacophore responsible for the molecular interaction with the GABA-A receptor (Figure 3). Next, we surveyed two major sites of structural modifications: molecular editing with fluorine around the phenyl ring and modification of the amide substituent at the alkyl tail. First, a fluorine atom was placed as a substitute for the methyl group at the four position of the phenyl ring, which is the group most prone to metabolic degradation.^7^ We envisioned that replacing this methyl group with incorporation of a fluorine atom might be harnessed to improve the metabolic stability of potential GABA-A receptor ligands. The value of this strategy steams out from our previous studies showing that single-site fluorination shows higher metabolic stability over compounds bearing two fluorine atoms attached at both sites of the aromatic system.^15,16^ Single-site fluorinations are frequently used to enhance the metabolic stability and bioavailability without disrupting molecular recognition with the biological target.^15−17^ We also reasoned that this substitution pattern would help to prevent potential epoxide formation within an aromatic system, which may lead to glutathione deprivation and hepatotoxicity. In addition, within the structural modifications performed around the aromatic system, we also decided to probe the tolerance of the methyl group incorporated into the 1*H*-benzo[*d*]imidazole core, changing the substitution pattern or replacing it with a hydrogen atom. The second important editing site included the substitution pattern around the amide function. We previously observed that aliphatic amides were well tolerated within this region and thus may be used to navigate the molecule into suitable regions of interaction between the α1/γ2 interface of the GABA-A receptor. Therefore, we envisioned that this site might be investigated within the 1*H*-benzo[*d*]imidazole series to expand the structure–activity relationship and identify the potential GABA-A receptor modulators.

**Scheme 1:**
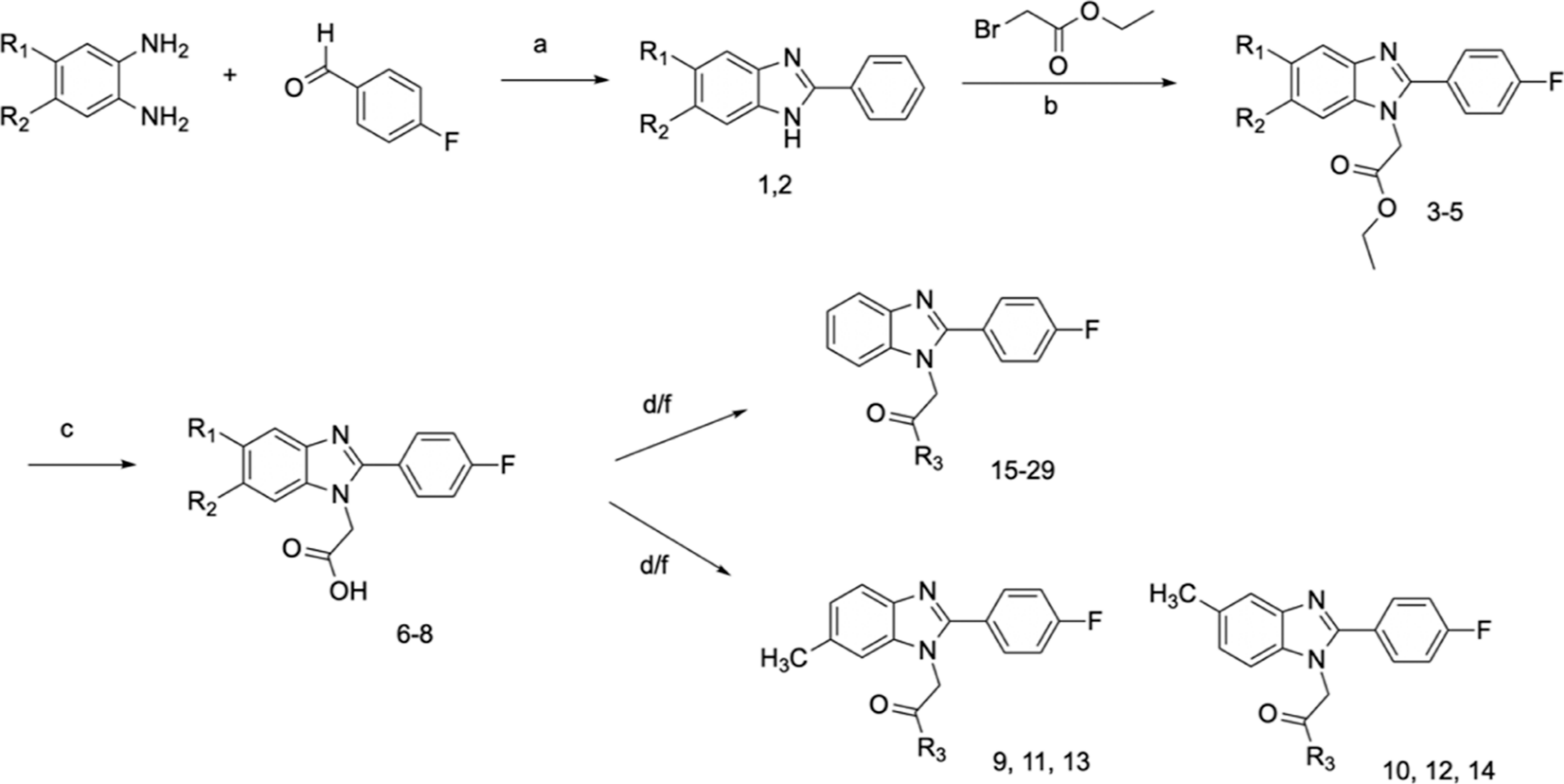
Reagents and conditions: (a) Na_2_S_2_O_5_, EtOH, H_2_O, 70 °C, and 24 h; (b) K_2_CO_3_, acetone, rt, and 12 h; (c) NaOH, EtOH, H_2_O, 70 °C, and 12 h; (d) TBTU, DIPEA, DCM, rt, 5 min then proper amine, 30 °C, 5 min, and microwaves; and (e) CDI, THF, 10 °C, 2 h then proper amine, 40 °C, and 12 h. R_1_, R_2_ = H, CH_3_, R_3_ = aliphatic amine, cyclic amine (see Table 1).

The library of 1*H*-benzo[*d*]imidazole series was prepared according to a three-step protocol which started with the condensation between the commercially available 4-fluorobenzaldehyde and the appropriate diamine to deliver **1** and **2** (Scheme 1). Treatment with ethyl 2-bromoacetate afforded intermediates **3**–**5**, which were further hydrolyzed to produce the key building blocks **6**–**8**. At this stage, compounds bearing methyl substituents (**4**, **5**, **7**, and **8**) were obtained as an inseparable mixture of regioisomers. The key building blocks were next reacted with propitiate amine in the presence of CDI in THF or TBTU and DIPEA in DCM to deliver the final compounds **9**–**29** (details in general chemistry information). In case of derivatives possessing methyl group in position 5 or 6, the synthesis delivered a mixture of regioisomers **9**, **11**, and **13** and **10**, **12**, and **14**, which were separated using a preparative HPLC system.

### 2.2 Structure–Activity Relationship and In Silico Studies

**Figure 4:**
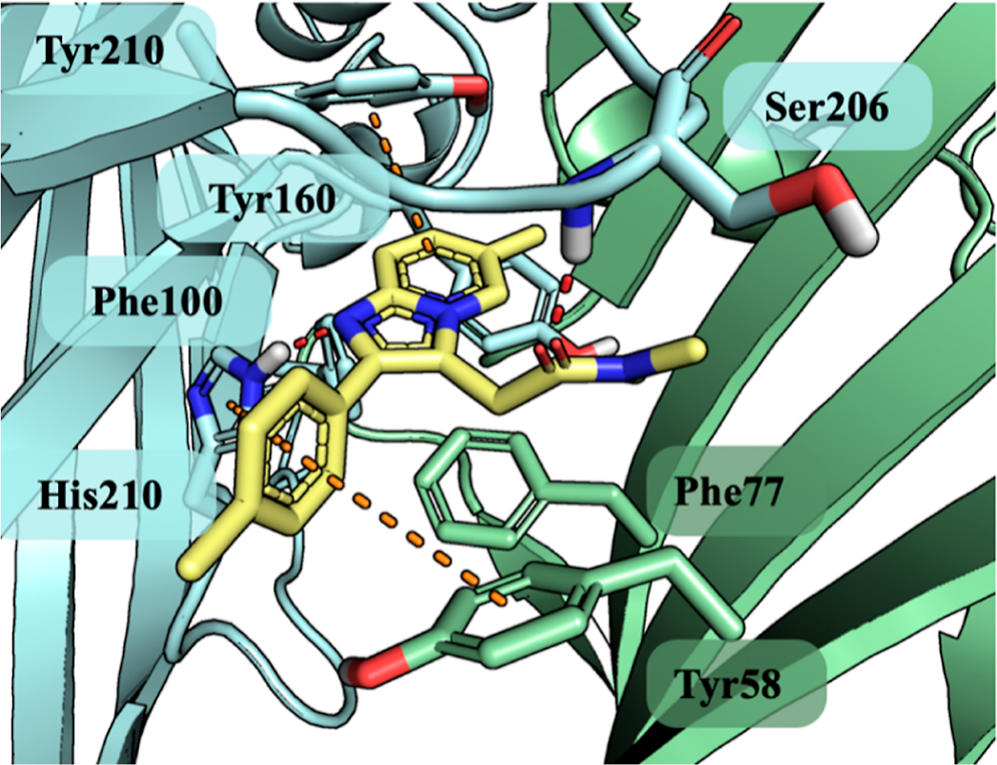
Zolpidem (yellow stick) docked to the benzodiazepine binding site of *h*GABA_A_. α1-subunit marked by cyan and the γ2-subunit by green color with interacting amino acids represented by sticks. Red dots present hydrogen bonds and orange presents the aromatic interaction, both CH−π and π–π.

All compounds were evaluated in radioligand binding studies measuring displacement capacities at the α1/γ2 interface of GABA-A receptors (benzodiazepine site). The results of the radioligand binding studies were complemented by molecular modeling studies to gain a deeper understanding of the specific interaction of 1*H*-benzo[*d*]imidazoles with the α1/γ2 interface of GABA-A receptors. First, we began with the in silico analysis of the binding pattern of a reference compound, zolpidem (p*K*_i_ = 7.39), to delineate structural elements that mediate molecular recognition at the allosteric recognition site of the human (α1β3γ2) *h*GABA-A receptor (benzodiazepine site, PDB ID—6HUP). The docking studies revealed that zolpidem located within the α1/2γ interface of the GABA-A receptor, forming the key hydrogen bond interactions with His102 of the α1-subunit and the Ser206 of γ2-subunit. The 2-phenylimidazo[1,2-*a*]pyridine core was anchored inside the aromatic pocket built with Phe100, His102, Tyr160, and Tyr210 of the α1-subunit and Phe77 and Tyr58 of the γ2-subunit and forms favorable aromatic interactions (Figure 4). All the interactions mentioned above within the α1/γ2 interface govern the allosteric action of zolpidem.^16,17^

Having this point of reference, we started to analyze the results obtained during the radioligand binding experiment. We observed that all compounds bearing the methyl group at the 6-position of the 1*H*-benzo[*d*]imidazole ring (**9**, **11**, and **13**) displayed the affinity for the GABA-A receptor, and the observed p*K*_i_ values were ranging from 5.1 to 5.53 (Table 1). The cyclic amides and aromatic amides were tolerated, given that the affinity values of these derivatives were almost identical. A subtle change in the location of methyl at the 1*H*-benzo[*d*]imidazole ring and placing it at the 5-position revealed to be detrimental to the activity of the GABA-A receptor, as compounds **10**, **12**, and **14** showed no affinity.

**Table 1:**
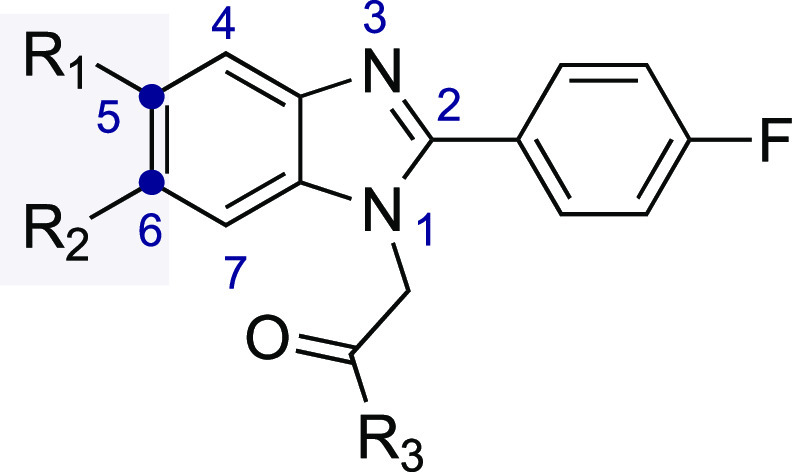

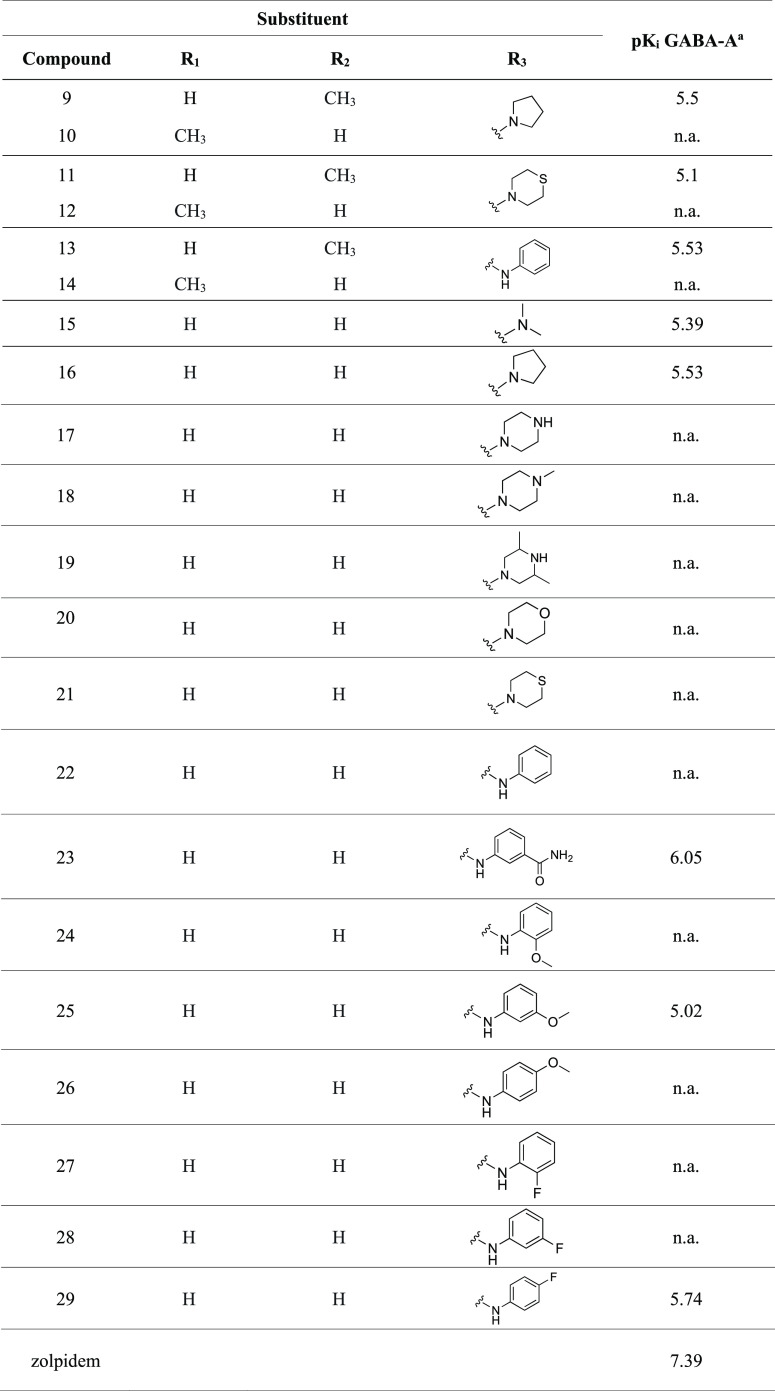
Structure and Binding Affinities for the GABA-A Receptor of Compounds (**9**–**29**)

a Binding affinity values are expressed as p*K*_i_ (i.e., −log *K*_i_) and expressed as mean ± SEM from at least three independent experiments achieved in duplicate. n.a.—no affinity.

**Figure 5:**
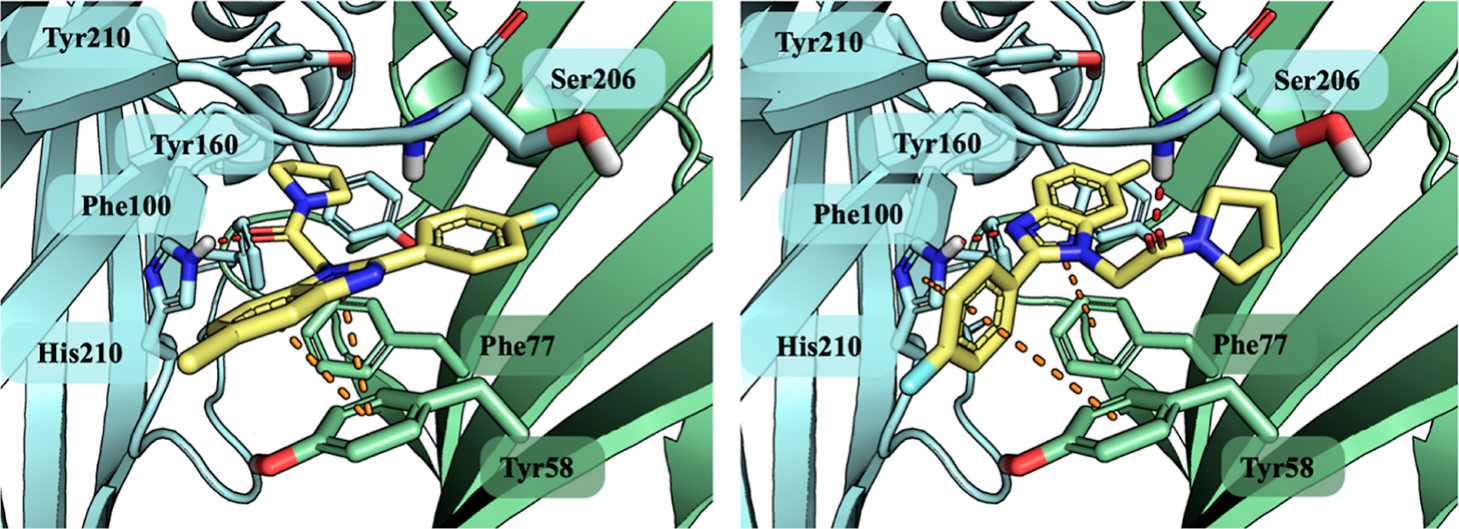
Compounds **10** (left panel) and **9** (right panel) docked to the benzodiazepine binding site of *h*GABA-A. Ligands represented by yellow sticks. α1-Subunit marked by cyan and γ2 by green colors with interacting amino acids represented by sticks. Red dots present hydrogen bonds and orange presents aromatic interaction, both CH−π and π–π.

In computational experiments, we observed that the position of the methyl group in the 1H-benzo [*d*]imidazole core influences the molecular recognition with the GABA-A receptor. The 6-methylbenzimidazole analogue **9** was found to be oriented similarly to zolpidem at the allosteric recognition site and reproduced crucial hydrogen bond interactions with αHis102 of the α1-subunit and γSer206 of the γ2-subunit. The 2-(4-fluorophenyl)-6-methyl-1*H*-benzo[*d*]imidazole ring was restrained in a pose similar to the 6-methyl-2-(*p*-tolyl) imidazo[1,2-*a*]pyridine core of zolpidem, where the nitrogen atom at position 3 of the 1H-benzo[*d*]imidazole system mimicked the interaction with αHis102 (Figure 5). The binding pose was stabilized by aromatic interactions with Phe100, His102, Tyr160, and Tyr210 of the α1-subunit and Phe77 and Tyr58 of the γ2-subunit. However, we observed fewer aromatic interactions compared to the reference ligand zolpidem (p*K*_i_ = 7.39), which may correspond to its lower activity (p*K*_i_ = 5.5).

Further, docking studies revealed that a subtle structural difference within the location of the methyl group and placing it at the 5-position of 1*H*-benzo[*d*]imidazole induce distinct changes of conformation within the allosteric recognition site. The 5-methylbenzimidazle ligand (**10**) adopts a divergent pose compared to the active ligands (**9**, zolpidem), and the molecule orientation is flipped within the α1/γ2 interface, posing a serious hindrance in the vicinity of the αAla161, αTyr160 containing loop. Moreover, the inverted ligand orientation precluded the formation of the crucial aromatic interaction with αPhe100, αHis102, and αTyr210 and impeded molecular recognition completely. The lack of these anchoring interactions results in a significant decrease in the reproducibility of the optimal binding pose and gave low values of the docking score, suggesting the possible explanation of the severe difference in affinity between **10** and **9**. Overall, these results suggest that the 2-(4-fluorophenyl)-6-methyl-1*H*-benzo[*d*]imidazole may be considered a suitable scaffold that directs molecular recognition with the GABA-A receptor at the α1/γ2 interface.

**Figure 6:**
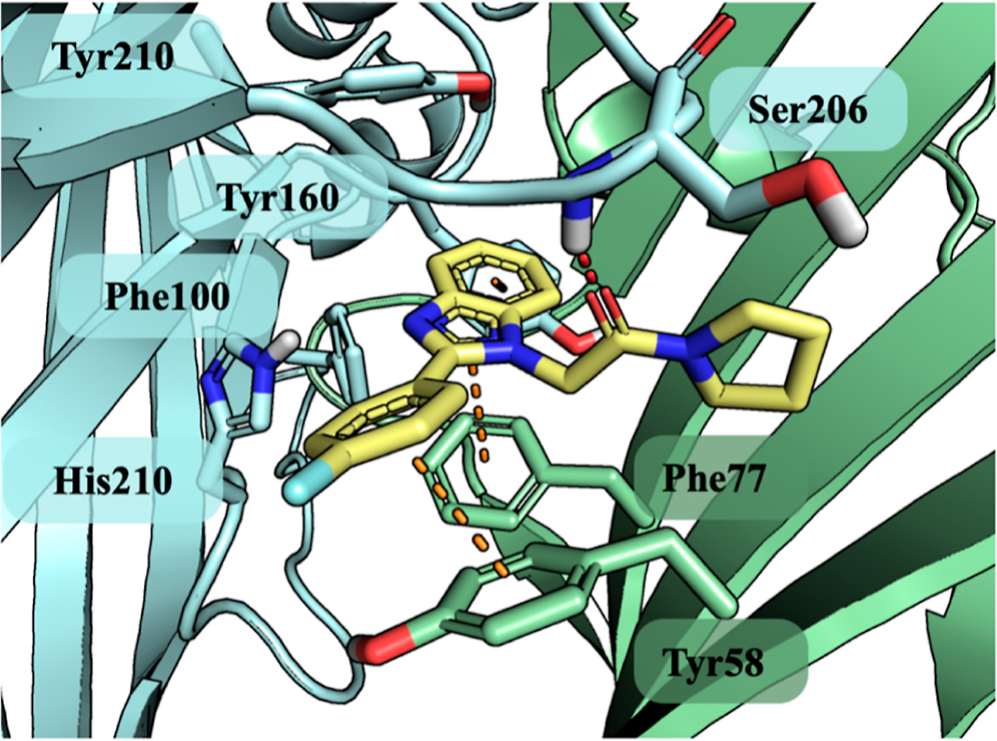
Compound **16** docked to the benzodiazepine binding site of *h*GABA-A. Ligands represented by yellow sticks. α1-Subunit marked by cyan and γ2 by green colors with interacting amino acids represented by sticks. Red dots present hydrogen bonds and orange presents the aromatic interaction, both CH−π and π–π.

Within a follow-up series, concerning 1*H*-benzo[*d*]imidazoles deprived of the methyl substituent (**15**–**29**), novel molecular recognition modes were observed. The binding patterns varied depending on amide functionality positioned in the amide chain. Compounds bearing dimethylamide (**15**) and pyrrolidine ring (**16**) conferred an advantageous interaction with the GABA-A receptor and elicited a reasonable binding affinity (p*K*_i_ = 5.39 and 5.53). However, replacement of the dimethylamide function with branched cyclic amines (e.g., piperazine **17** or morpholine **20**) resulted in a loss of activity. Substitution patterns of derivatives bearing aromatic amides indicated that meta position functional groups play a role in binding with the GABA-A receptor. To gain further insight into molecular interactions between the GABA-A receptor and 2-(4-fluorophenyl)-1*H*-benzo[*d*]imidazoles deprived of the methyl substituent, we docked **16** and **23**. The 2-(4-fluorophenyl)-1*H*-benzo[*d*]imidazole analogue bearing a pyrrolidine ring (**16**) in the amide tail organized the allosteric recognition site in a similar way to zolpidem (Figure 6). We observed formation of key hydrogen bonds with His102 and Ser206 at the α1/γ2 interface. However, the lack of a methyl group in the 1*H*-benzo[*d*]imidazole scaffold contributed to the poorer alignment of the three-ring system, which clearly resulted in deterioration of the aromatic interaction with αPhe100, αHis102, αTyr160, and αTyr210 and γPhe77 and γTyr58. This in turn may trigger the observed drop in activity (p*K*_i_ = 5.53) of **16**.

Compounds endowed with the 3-aminobenzamide (**23**) or 3-methoxyaniline (**25**) motif, provided interaction with the GABA-A receptor with p*K*_i_ values of 6.05 (**23**) and 5.02 (**25**). However, the swapping of the methoxy group position to orto (**24**) or para (**26**) resulted in a loss of affinity, suggesting that the position of the substituent in the aniline ring could be an additional prerequisite for relevance in molecular recognition with the target. Alongside, we observed that the fluorine atom positioned in the para position of aniline (**29**) interacted with the GABA-A receptor with p*K*_i_ = 5.74, suggesting that additional favorable interactions might be formed in this position. However, shifting the substitution pattern of the fluorine atom into orto or meta resulted in a decrease in affinity, delineating the importance of the relative position of fluorine in molecular recognition. Investigation of a binding pose of compounds possessing a benzamide moiety (**23**) and 4-fluoroaniline (**29**) incorporated in the amide function showed that these compounds were able to restore the binding patterns of active compounds. The 3-aminobenzamide moiety formed an additional hydrogen bond interaction in the vicinity to γAsp56, which is not present in the case of **29**, bearing a 4-fluorophenyl fragment. Despite this difference, in both cases, the compounds form a strong network of interactions with the α1/γ2 interface of the GABA-A receptor.

### 2.3 Electrophysiological Studies

Given that ligands binding within the α1/γ2 interface act as PAMs, we selected the most promising 1*H*-benzo[*d*]imidazoles (**9**, **15**, **16**, **23**, and **29**) for electrophysiological studies using automated patch clamp recordings (QPatch16X, Sophion). We expected that 1*H*-benzo[*d*]imidazoles would potentiate GABA-induced Cl^–^ currents rather than inducing ion passage alone. Indeed, when surveyed alone, the tested molecules did not induce a change in ion currents, suggesting a lack of agonistic properties. In contrast, in the presence of 10 μM of GABA, we observed a significant increase in GABA-induced currents. This experiment demonstrated that all tested 1*H*-benzo[*d*]imidazoles were effective in enhancing the GABA-induced ion currents, confirming their allosteric modulatory properties. In particular, compound **9** notably increased the GABA-induced Cl^–^ currents (172% of the GABA efficacy), being the most effective derivative of 1*H*-benzo[*d*]imidazoles (Table 2). The remaining compounds **16** and **23** also performed well in this assay, showing allosteric modulatory efficacy (124 and 132% of GABA efficacy), while compounds **15** and **29** displayed weak modulatory properties (**15**: 105% and **29**: 112% of GABA efficacy). Although we did not observe a direct correlation between binding potencies and functional effects, one has to consider that ion gating by allosteric modulators is a complex endeavor that relies on various factors. The acquired findings suggest that compounds can facilitate channel opening by inducing a specific conformational change of the GABA-A receptor, which we observe in functional responses. Based on the abovementioned results, we selected the most promising compounds **9** and **23** for further studies.

**Table 2:** Evaluation of Positive Allosteric Modulation (PAM) Properties for Selected Compounds in HEK293 Cells Stably Expressing α1β2γ2-GABA-A Receptors

compound	% of 10 μM GABA response^a^
**9**	172 ± 12,5
**15**	105 ± 2,5
**16**	124 ± 5,2
**23**	132 ± 13,2
**29**	112 ± 2,8
zolpidem	212 ± 8,5

a Results presented as a fold increase of GABA-gated current amplitude compared to the efficacy of 10 μM γ-aminobutyric acid (GABA) alone. Molecules were tested in 1 μM concentration. Data represent a mean ± SEM of at least three experiments performed on distinct cells.

### 2.4 Thermodynamic Solubility in pH = 7.4

Prior to cellular studies, we determined the solubility of **9** and **23**, given that poor compound solubility may impact the biological data quality.^18^ Therefore, we measured thermodynamic solubility of **9** and **23** in phosphate buffer of pH = 7.4, using perphenazine as a reference compound. According to early drug discovery criteria,^18^ compound **23** displayed favorable solubility of 74.00 ± 0.81 μg/mL, while compound **9** revealed a moderate solubility (50.00 ± 0.54 μg/mL). These values are considered as acceptable and enable us to proceed with in cellular experiments.

### 2.5 Metabolic Stability and Hepatotoxicity

Given that our goal was to obtain metabolically stable scaffolds, we selected **9** and **23** as representatives of each series for metabolic stability studies using human liver microsomes (HLMs). In parallel, we interrogated the quality of 1*H*-benzo[*d*]imidazole derivatives by confronting them head-to-head with the comparator drug alpidem. The latter compound undergoes rapid biotransformation forming a reactive epoxide intermediate that induces glutathione deprivation and acute liver toxicity in patients.^7,19,20^

In line with the literature data,^19^ we observed that alpidem was heavily metabolized producing high content of metabolites (M1, M2, M3, M4, and M5), and solely 38.60% of the parent compound remained unmetabolized (Table 3), after 120 min of incubation with HLMs. Literature data suggest that the biotransformation pathway of alpidem occurs mainly within 7- and 8-position of the imidazo[1,2-*a*]pyridine ring, producing an epoxide intermediate, which is further transformed into hydroxy counterparts or glutathione adducts (Figure 2).^19^ Although we did not detect an epoxide intermediate, we detected two hydroxylated metabolites (M1 and M2), which suggest that the epoxy intermediate may undergo rapid transformation to final hydroxy metabolites (Table 4 and Figures S1 and S2—Supporting Information). Alongside, we observed that the dipropylamide moiety was also highly vulnerable to metabolic degradation and the remaining biotransformation pathway included dealkylation and hydroxylation (M3 and M5).

**Table 3:** Thermodynamic Solubility of **9** and **23** in Phosphate Buffer pH = 7.4

compound	aqueous solubility μg/mL
**9**	50.00 ± 0.54
**23**	74.00 ± 0.81
perphenazine	30.60 ± 0.32

**Table 4:** Metabolic Stability/Biotransformation Pathways in the Presence of HLMs

compound	molecular mass (*m*/*z*) +H^+^	% remaining	molecular mass of the metabolite (*m*/*z*)	metabolic pathway^a^
**alpidem**	404.22	38.60	420.24 (M1)	hydroxylation^b^
			420.24 (M2)	hydroxylation
			378.10 (M3)	dealkylation and hydroxylation
			418.24 (M4)	oxidation
			378.17 (M5)	dealkylation and hydroxylation
**23**	389.27	89.13	405.22 (M1)	hydroxylation^b^
**9**	338.23	90.12	354.18 (M1)	hydroxylation^b^
			354.18 (M2)	hydroxylation
			370.20 (M3)	double hydroxylation

a The metabolic stability of the tested compounds was evaluated in the presence of HLMs with the percentage of the parent compound remaining measured after 120 min of incubation with HTMs.

b The main metabolic pathway.

Conversely, the 1*H*-benzo[*d*]imidazole probes displayed higher metabolic stability compared to alpidem, which was evident from the high amounts of parent compounds that did not undergo metabolic degradation (Table 3). This was true for the 2-(4-fluorophenyl)-6-methyl-1*H*-benzo[*d*]imidazole derivative **9**, which remained unmetabolized at 90%, after 120 min of incubation. As predicted by MetaSite software, the main metabolic pathway involved hydroxylation, possibly within the methyl group and the aromatic ring (M1 and M2) or double hydroxylation (M3) (Figures S3 and S4—Supporting Information). These results suggest that single-site CH_3_ → F-substitution at the phenyl termini leads to enhanced metabolic stability. In agreement with this trend, our previous reports pointed to a similar conclusion that metabolic stability could be enhanced with single-site fluorination in terms of the 4-phenyl position of phenylimidazo[1,2-*a*]pyridine.^15^ In contrast, insertion of fluorine atoms at 4-phenyl and 6-imidazo[1,2-*a*]pyridine position triggers rapid metabolic biotransformation, resulting in compounds with low metabolic stability.^16^ This is also evident in the case of 6-chloro-2-(4-chlorophenyl)imidazo[1,2-*a*]pyridine derivatives such as alpidem, where placing chloride atoms at both furthest positions of the 2-phenylimidazo [1,2-*a*] pyridine system induce rapid metabolic degradation.^19^

**Figure 7:**
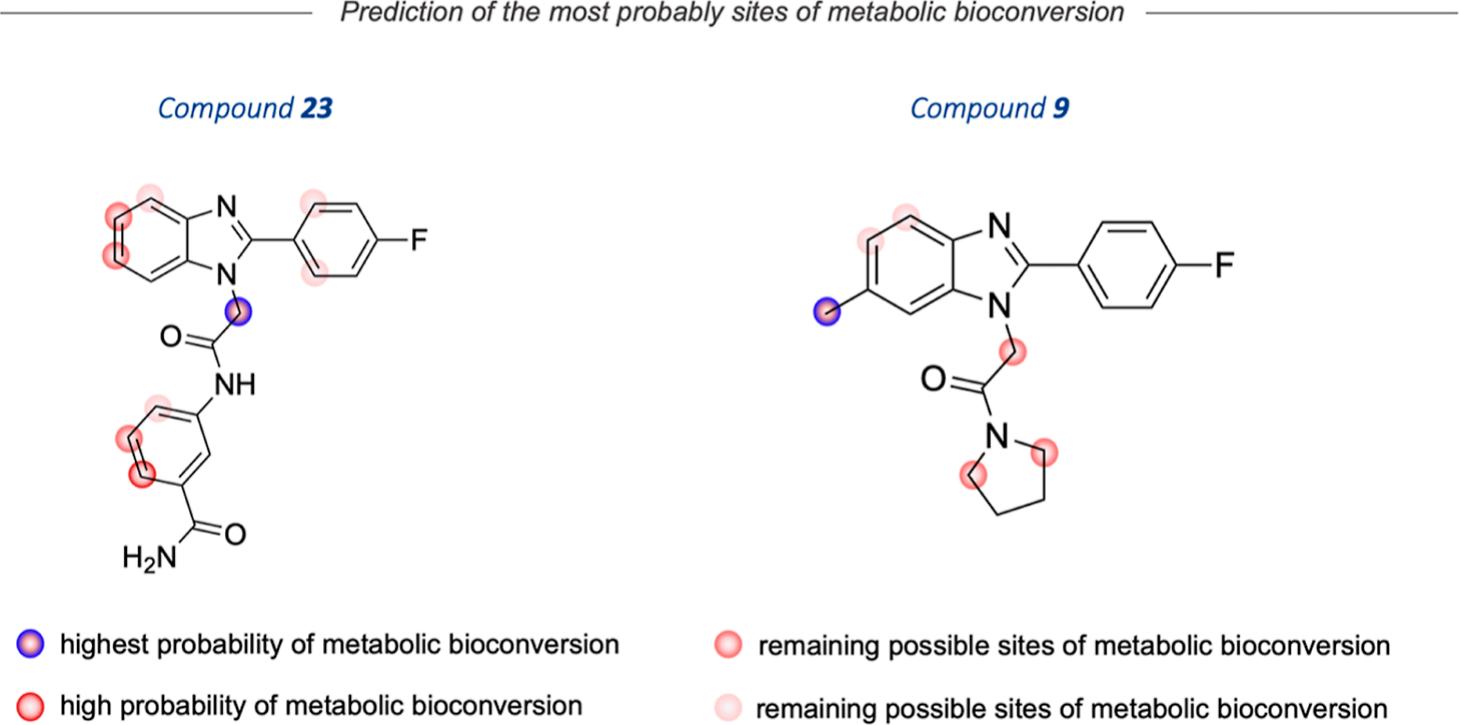
MetaSite 6.0.1. Software prediction of the most probable sites of **23** and **9** metabolism.

The 2-(4-fluorophenyl)-1*H*-benzo[*d*]imidazole derivative **23** also showed to be less prone to metabolic degradation, as 89.13% of the parent compound is unchanged under the same experimental conditions (Figures S5 and S6—Supporting Information). We observed the formation of a single hydroxylated metabolite (M1) in 10.87%. The MetaSite software identified the aliphatic chain as a possible site of degradation (Figure 7). However, considering that hydroxylation in this position would lead to the formation of an unstable hemiaminal and degradation of the molecule, it is possible that the primary metabolic transformation occurs within aromatic rings, producing stable hydroxylated metabolites. Overall, these results indicate that the 2-(4-fluorophenyl)-1*H*-benzo[*d*]imidazole scaffold may help to generate compounds with suitable metabolic stability.

**Figure 8:**
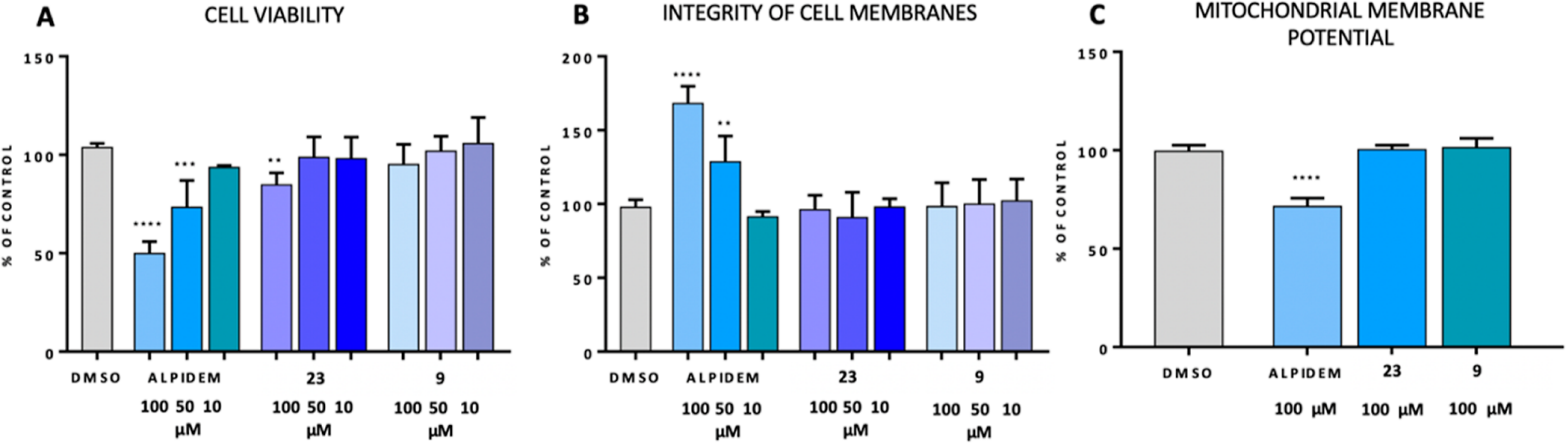
Hepatotoxicity assays for selected lead molecules. Cell viability (A) was measured using the PrestoBlue reagent, cell membrane damage (B) was assessed using the ToxiLight bioassay, and mitochondrial membrane potential (C) was assessed using a JC-1 probe. Assays performed after 24 h treatment with tested compounds and a reference alpidem. Results are presented as mean % of untreated controls ± S.D. (*n* = 3). Differences among groups were evaluated by one-way ANOVA followed by post-hoc analysis (Dunnett’s multiple comparison tests) and were considered statistically significant if *p* < 0.05 (****p < 0.0001, ***p < 0.001, **p < 0.01, and *p < 0.05).

Given that drug-induced liver injury (DILI) is the leading cause of the termination of potential drug candidates,^21,22^ two of the main molecules **9** and **23** were further assayed in HepG2 human hepatoma cells to assess their potential risk of toxicity.^23^ The hepatotoxicity of the compounds tested was evaluated using two methods: cell viability (Figure 8A,B) and mitochondrial membrane potential assay (Figure 8C). Both tested molecules **9** and **23** did not induce any significant cytotoxic effect at concentrations up to 50 μM (Figure 8), indicating a promising safety range. Similarly, we did not observe mitochondrial membrane potential (MMP) attenuation of HepG2 cells, after treatment with **9** and **23**, tested in 100 μM (Figure 8C). The assays were performed in parallel with alpidem, a known inducer of hepatotoxicity, which in these settings showed noticeable hepatotoxicity at 100 and 50 μM and induced mitochondrial dysfunction at 100 μM (Figure 8). Overall, these results suggest that the hepatotoxic effects may be lessened by modulation of the chemical structure.

## 3 Conclusions

The interest in developing PAMs of GABA-A receptors has been fueled by clinical findings, which revealed that modulation of α1β2γ2 GABA-A receptors in the basal ganglia region can be used to mitigate neurological dysfunctions.^3,4^ Despite the compelling evidence provided by clinical data on the utility of this strategy, the current chemical space of molecules capable of preferential binding within the α1/γ2 allosteric recognition site at the GABA-A receptor is limited to imidazo[1,2-*a*]pyridine derivatives that undergo rapid biotransformation. These findings prompted us to expand the available chemical space and design a series of 2-(4-fluorophenyl)-1*H*-benzo[*d*]imidazoles with the goal of identifying new scaffolds that omit high metabolic vulnerability and therefore exert positive allosteric modulatory properties at GABA-A receptors. In this regard, we applied single-site CH_3_ → F replacement to enhance metabolic stability without affecting allosteric recognition site affinity at the GABA-A receptor. Therefore, we synthesized a library of 2-(4-fluorophenyl)-1*H*-benzo[*d*]imidazoles that were investigated in radioligand binding studies to verify their biding capacities at the GABA-A receptor. We observed that 2-(4-fluorophenyl)-1*H*-benzo[*d*]imidazoles mimic the steroelectronic properties of the imidazo[1,2-*a*]pyridine scaffold and govern molecular recognition with the GABA-A receptor at the α1/γ2 interface. Furthermore, our studies showed that placing the methyl group at the 6-position of the 1*H*-benzo[*d*]imidazole scaffold navigates the molecule to the region of the allosteric recognition site, while the 5-methyl derivative induced a steric penalty and abolished the interaction with the GABA-A receptor. The deprivation of the methyl group on the 2-(4-fluorophenyl)-1*H*-benzo[*d*]imidazole scaffold also influenced the molecular interaction with the GABA-A receptor, although it could be compensated by placing a suitable substituent at the amide alkyl chain. This led us to select the most active derivatives of 2-(4-fluorophenyl)-6-methyl-1*H*-benzo[*d*]imidazole (**9**) and 1*H*-benzo[*d*]imidazole (**23**) for further biological investigation. Both compounds were characterized by acceptable aqueous solubility in pH = 7.4 (50 and 74 μg/mL). In electrophysiological studies, we observed that novel scaffolds were effective in enhancing the GABA-induced ion currents, confirming their allosteric modulatory properties. Further cellular studies showed that single-site fluorination at the 4 position of the phenyl ring in the 2-phenyl-1*H*-benzo[*d*]imidazole helps to enhance the metabolic stability without influencing molecular recognition with the GABA-A receptor. The present study points to 2-(4-fluorophenyl)-1*H*-benzo[*d*]imidazole and 2-(4-fluorophenyl)-6-methyl-1*H*-benzo[*d*]imidazole as suitable scaffolds in the acquisition of PAMs of the GABA-A receptor with enhanced metabolic stability and reduced potential for hepatotoxicity, which makes them potential lead structures for further investigation in the field of neuropharmacology research. The most promising compounds selected in this study, **9** and **23**, may serve as tool compounds to probe the activity of GABA-A receptors, including those expressed in the basal ganglia region with therapeutic potential in various neurological dysfunctions related to basal ganglia pathologies.

## 4 Experimental Section

### 4.1 In Silico Methods

All tested and reference compounds were prepared with the Maestro (Schrödinger) software. The LigPrep tool was used to calculate the partial charges (pH 7.4 ± 0.2), atom types, and check geometry of the ligands. The *h*GABA-A receptor complex with diazepam available in the PDB database (Protein Data Base) under 6HUP code was used in docking studies. It contains full-chain (α1β3γ2) *h*GABAA heterodimers obtained by cryoelectron microscopy. The protein complex was prepared using the ProteinPreparation Wizard tool (Maestro-Schrödinger). Appropriate charges were assigned, and the geometry of amino acid and the hydrogen bond network was adjusted (for pH 7.4).

The binding site was optimized to better reflect the interactions with imidazopyridine derivatives. To achieve this, the docking (Glide, Maestro-Schrödinger) of the reference compound library (Supporting Information) was performed. Among the obtained results, the most common conformation observed among active compounds which present interactions with key amino acids described in the literature was selected. Such complexes were minimized using the Refine Protein–Ligand Complex protocol available from Maestro-Schrödinger (25 steps of optimization with the Monte Carlo protocol for all amino acids in 10 Å radius from the ligand). Docking and optimization processes were repeated five times until stable and reproducible results were obtained for zolpidem and other active imidazopyridine derivatives.

Optimized complexes were used for docking studies on tested compounds. The binding site was defined as a cube with the center at the imidazopyridine derivatives and size optimal for ligands with length equal to 25 Å or less. The XP (extra precision) docking protocol of Glide (Maestro-Schrödinger) was applied. Additionally, during the calculations, we considered the presence of H-aromatic bonds and the intramolecular hydrogen bonds. For each ligand, the highest rated pose and an assessment of interaction with individual amino acids within 12 Å from the grid center were collected. The docking was performed three separate times to verify the repeatability of the obtained results. The results were visualized using the PyMol program (Schrödinger).

### 4.2 Synthesis

#### 4.2.1 General Chemistry Information

All chemicals were purchased as reagent grade and used directly without further purification. Flash chromatography was performed using a CombiFlash RF (Teledyne Isco) and single-use silica gel flash columns RediSep Rf (silica gel 60, particle size 40–63 μm) and RediSep Gold (silica gel 60, particle size 20–40 μm) purchased from Teledyne Isco. Reactions were monitored by thin-layer chromatography (TLC) on aluminum foil precoated with silica gel 60 F_254_ (Merck). The reaction mixtures were visualized with UV light (254 nm). Ultraperformance liquid chromatography (UPLC)–mass spectrometry (MS) or UPLC—tandem mass spectrometry (MS/MS) analysis was performed on a UPLC–MS/MS system containing a Waters ACQUITY UPLC (Waters Corporation, Milford, MA, USA) coupled with a Waters tandem quadrupole mass spectrometer (TQD) (electrospray ionization (ESI) mode with TQD). Chromatographic analyses were performed using the ACQUITY UPLC BEH (bridged ethyl hybrid) C18 column: particle size of 2.1 × 100 mm and 1.7 μm particle size. The column was eluted under gradient conditions using 95–0% eluent A for 10 min at a flow rate of 0.3 mL/min, at 40 °C. Eluent A: 0.1% solution of formic acid in water (v/v); eluent B: 0.1% solution of formic acid in acetonitrile (v/v). Injection volume: 10 μL of each sample and chromatograms were recorded using a Waters eλ photodiode array detector. The spectra were analyzed in the range of 200–700 nm with a resolution of 1.2 nm and at a sampling rate of 20 points/s. The UPLC–MS purity of all the compounds submitted to biological assays were determined to be 95–100%. Preparative HPLC was performed with a Jasco BS-4000-1, equipped with Luna 5 μm C8(2) 100 Å, LC Column 150 × 21.2 mm column. The mobile phase was composed of acetonitrile + 0.05% formic acid and water + 0.05% formic acid. The ^1^H NMR, ^13^C NMR, and ^19^F NMR spectra were recorded on a FT-NMR 500 MHz JEOL spectrometer (JEOL Ltd., Tokyo, Japan) JNM-ECZR500 RS1, version ECZR in CHLOROFORM-d, METHANOL-*d*_4_, or DMSO-*d*_6_ operating at 500 MHz (^1^H NMR), 126 MHz (^13^C NMR), and 471 MHz (^19^F NMR), respectively. The *J* values are expressed in hertz (Hz). Chemical shifts, δ, are expressed in ppm and reported taking reference of the appropriate deuterated solvent. Signal multiplicities are described using the following abbreviations: s (singlet), br s (broad singlet), bd (broad doublet), d (doublet), dd (doublet of doublets), dt (doublet of triplets), t (triplet), td (triplet of doublets), tdd (triplet of doublet of doublets), q (quartet), dq (doublet of quartets), quint (quinted), and m (multiplet). NMR spectra were analyzed using the ACD/Spectrus Processor 2017. The LCMS spectra were analyzed using Waters MassLynx 4.0 software. The melting points were measured using the Büchi Melting Point B-540 apparatus in open glass capillaries and are not corrected.

#### 4.2.2 General Procedure for the Preparation of Key Intermediates **1** and **2**

A mixture of appropriate aromatic diamine (1 equiv) and 4-fluorobenzaldehyde (1 equiv) in ethanol (1 mL/mmol) was mixed with a 5 M aqueous solution of sodium metabisulfate (1.5 equiv). The mixture was stirred at 70 °C for 24 h. After that time, the reaction mixture was cooled to room temperature, and a portion of distilled water was added (2× reaction mixture volume). Next, the mixture was cooled to a temperature of 0 °C and kept at this temperature for 2 h until the precipitate was formed. The solid was filtrated and washed with distilled water, and the solvent was evaporated under reduced pressure. The crude mixture was purified by flash column chromatography over silica gel using dichloromethane/diethyl ether/methanol, 70:29:1 (v/v/v) as eluent.

#### 4.2.3 2-(4-Fluorophenyl)-1*H*-benzo[*d*]imidazole (**1**)

The title compound was obtained using 1,2-phenylenediamine (1 equiv, 10.0 mmol, 1.080 g) and 4-fluorobenzaldehyde (1 equiv, 10.0 mmol, 1.241 g) in ethanol (10 mL) and sodium metabisulfate (1.5 eq, 15.0 mmol, 2.852 g) in water (3 mL). Column chromatography purification using dichloromethane/diethyl ether/methanol 70:29:1 (v/v/v) afforded **1** as a white powder (92% yield). *R*_*f*_ (dichloromethane/diethyl ether/methanol 7:2.9:0.1) = 0.65. ^1^H NMR (500 MHz, DMSO-*d*_6_, 295 K): δ (ppm) 12.89 (br s, 1H), 8.21–8.16 (m, 2H), 7.56 (br s, 2H), 7.39–7.34 (m, 2H), 7.20–7.14 (m, 2H). ^13^C NMR (126 MHz, DMSO-*d*_6_, 295 K): δ (ppm) 163.59 (d, *J* = 246.87 Hz), 150.92, 141.80, 136.12, 129.25 (d, *J* = 8.45 Hz), 127.34 (d, *J* = 3.02 Hz), 122.86, 116.54 (d, *J* = 21.73 Hz), aromatic carbons overlap. ^19^F NMR (471 MHz, DMSO-*d*_6_, 295 K): δ (ppm) −111.04 (s, 1F). LC–MS (ESI) calcd for C_14_H_11_FN_2_, 213.078 [M + H^+^]; found, 213.039 [M + H^+^].

#### 4.2.4 2-(4-Fluorophenyl)-6-methyl-1*H*-benzo[*d*]imidazole (**2**)

The title compound was obtained using 4-methylbenzene-1,2-diamine (1 equiv, 10.0 mmol, 1.222 g) and 4-fluorobenzaldehyde (1 equiv, 10.0 mmol, 1.241 g) in ethanol (10 mL) and sodium metabisulfate (1.5 equiv, 15.0 mmol, 2.852 g) in water (3 mL). Column chromatography purification using dichloromethane/diethyl ether/methanol 70:29:1 (v/v/v) afforded **2** as a creamy powder (92% yield). *R*_*f*_ = 0.65 (dichloromethane/diethyl ether/methanol, 7.0/2.9/0.1, v/v/v). ^1^H NMR (500 MHz, CD_3_OD, 294 K): δ (ppm) 8.09–7.95 (m, 2H), 7.45 (d, *J* = 8.3 Hz, 1H), 7.39–7.31 (m, 1H), 7.29–7.16 (m, 2H), 7.12–7.04 (m, 1H), 2.41–2.37 (m, 3H). ^13^C NMR (126 MHz, CD_3_OD, 295 K): δ (ppm) 164.44 (d, *J* = 246.87 Hz), 149.98, 137.00, 135.46, 133.7, 129.2 (d, *J* = 3.02 Hz), 125.02, 124.47, 115.9 (d, *J* = 8.45 Hz), 113.8 (d, *J* = 21.73 Hz), 20.39. ^19^F NMR (471 MHz, CD_3_OD, 294 K): δ (ppm) −110.83 (s, 1F). LC–MS (ESI) calcd for C_14_H_11_FN_2_, 227.094 [M + H^+^], 227.039 [M + H^+^].

#### 4.2.5 General Procedure for the Preparation of Key Intermediates **3–5**

A mixture of an appropriate intermediate **1** or **2** (1 equiv) and potassium carbonate (2 equiv) in anhydrous acetone (3.26 mL/mmol) was stirred at room temperature for 2 h. After that time, ethyl bromoacetate (1 equiv) was added, and the mixture was stirred at room temperature for another 12 h. Next, potassium carbonate was filtered off, acetone was evaporated, and the crude mixture was purified by “flash” column chromatography over silica gel using dichloromethane/diethyl ether/methanol 70:29:1 (v/v/v) as an eluent.

#### 4.2.6 Ethyl 2-(2-(4-Fluorophenyl)-*1H*-benzo[*d*]imidazole-1-yl)acetate (**3**)

The title compound was obtained using 2-(4-fluorophenyl)-1*H*-benzo[*d*]imidazole (**1**) (1 eq, 9.20 mmol, 1.950 g), ethyl bromoacetate (1 eq, 9.20 mmol, 1.536 g), potassium carbonate (2 eq, 18.40 mmol, 2.539 g), and anhydrous acetone (30 mL). Column chromatography purification using dichloromethane/diethyl ether/methanol 70:29:1 (v/v/v) afforded **3** as a white powder (95% yield). *R*_*f*_ (dichloromethane/diethyl ether/methanol 7:2.9:0.1) = 0.71. ^1^H NMR (500 MHz, DMSO-*d*_6_, 295 K): δ (ppm) 7.77–7.70 (m, 2H), 7.69–7.64 (m, 1H), 7.58–7.53 (m, 1H), 7.41–7.33 (m, 2H), 7.25 (m, 2H), 5.19 (s, 2H), 4.07 (q, *J* = 7.1 Hz, 2H), 1.09 (t, *J* = 7.2 Hz, 3H). ^13^C NMR (126 MHz, DMSO-*d*_6_, 295 K): δ (ppm) 168.74, 163.51 (d, *J* = 247.47 Hz), 152.92, 142.82, 136.77, 131.86 (d, *J* = 9.05 Hz), 126.97 (d, *J* = 3.02 Hz), 123.35, 122.89, 119.71, 116.47 (d, *J* = 21.73 Hz), 111.18, aromatic carbons overlap, 61.92, 46.48, 14.43. ^19^F NMR (471 MHz, DMSO-*d*_6_, 295 K): δ (ppm) −110.85 (s, 1F). LC–MS (ESI) calcd for C_17_H_15_FN_2_O_2_, 299.115 [M + H^+^]; found, 299.250 [M + H^+^].

#### 4.2.7 Ethyl 2-(2-(4-Fluorophenyl)-5-methyl-1*H*-benzo[*d*]imidazole-1-yl)acetate (**4**) and Ethyl 2-(2-(4-Fluorophenyl)-6-methyl-1*H*-benzo[*d*]imidazole-1-yl)acetate (**5**)

The title compounds (**4**) and (**5**) were obtained as an inseparable mixture of regioisomers using 2-(4-fluorophenyl)-6-methyl-1*H*-benzo[*d*]imidazole (**2**) (1 equiv, 9.20 mmol, 1.950 g), ethyl bromoacetate (1 equiv, 9.20 mmol, 1.536 g), potassium carbonate (2 equiv, 18.40 mmol, 2.539 g), and anhydrous acetone (30 mL). Compounds were further used without purification. *R*_*f*_ (dichloromethane/diethyl ether/methanol 7:2.9:0.1) = 0.71. LC–MS (ESI) calcd for C_18_H_17_FN_2_O_2_, 313.131 [M + H^+^]; found, 313.250 [M + H^+^].

#### 4.2.8 General Procedure for the Preparation of Key Intermediates (**6–8**)

Appropriate intermediates **3–5** (1 equiv) were dissolved in ethanol 98% (1.2 mL/mmol). Next, 1 M NaOH aqueous solution (1.2 mL/mmol) was added. The reaction mixture was stirred at 70 °C for 12 h. After that time, the mixture was cooled to the room temperature. Next, 1 M hydrochloric acid was added until the pH of 4.6. Then, the solid product was filtered, washed with cold distilled water, and dried. The crude product was then purified by crystallization from methanol.

#### 4.2.9 2-(2-(4-Fluorophenyl)-1*H*-benzo[*d*]imidazole-1-yl)acetic Acid (**6**)

The title compound was obtained using ethyl 2-(2-(4-fluorophenyl)-1*H*-benzo[*d*]imidazole-1-yl)acetate (**3**) (1 equiv, 8.33 mmol, 2.482 g), ethanol (10 mL), and a 1 M aqueous solution of NaOH (10 mL). Crystallization from methanol afforded **6** as a white powder with 55% yield. *R*_*f*_ (dichloromethane/methanol 7:3) = 0.85. ^1^H NMR (500 MHz, DMSO-*d*_6_, 295 K): δ (ppm) 7.76–7.71 (m, 2H), 7.68–7.64 (m, 1H), 7.57–7.53 (m, 1H), 7.41–7.35 (m, 2H), 7.28–7.20 (m, 2H), 5.06 (s, 2H). ^13^C NMR (126 MHz, DMSO-*d*_6_, 295 K): δ (ppm) ppm 170.19, 163.49 (d, *J* = 247.47 Hz), 152.92, 142.79, 136.83, 131.83 (d, *J* = 9.05 Hz), 127.09 (d, *J* = 3.02 Hz), 123.27, 122.78, 119.61, 116.46 (d, *J* = 21.73 Hz), 111.21, aromatic carbons overlap, 46.51. ^19^F NMR (471 MHz, DMSO-*d*_6_, 295 K): δ (ppm) −110.91 (s, 1F). LC–MS (ESI) calcd for C_15_H_11_FN_2_O_2_, 271.084 [M + H^+^]; found, 271.380 [M + H^+^].

#### 4.2.10 2-(2-(4-Fluorophenyl)-5-methyl-1*H*-benzo[*d*]imidazole-1-yl)acetic Acid (**7**) and 2-(2-(4-Fluorophenyl)-6-methyl-1*H*-benzo[*d*]imidazole-1-yl)acetic Acid (**8**)

The title compounds (**7**) and (**8**) were obtained as an inseparable mixture of regioisomers. The mixture of the title compounds was obtained using a mixture of ethyl 2-(2-(4-fluorophenyl)-5-methyl-1*H*-benzo[*d*]imidazole-1-yl)acetate (**4**) and ethyl 2-(2-(4-fluorophenyl)-6-methyl-1*H*-benzo[*d*]imidazole-1-yl)acetate (**5**) (1 equiv, 8.33 mmol, 2.600 g), ethanol (10 mL), and a 1 M aqueous solution of NaOH (10 mL). Crystallization from methanol afforded an inseparable mixture of regioisomers, **6** and **7** as a white powder (50% yield), that were used directly to the next step without further purification. LC–MS (ESI) calcd for C_16_H_13_FN_2_O_2_, 285.099 [M + H^+^]; found, 285.38 [M + H^+^].

#### 4.2.11 General Procedure for the Preparation of Final Compounds (**9–12**)

The mixture of intermediates **7** and **8** (1 equiv, 0.70 mmol, 0.200 g) was dissolved in dichloromethane (4.29 mL/mmol). Then, TBTU (2 equiv, 1.40 mmol, 0.228 g) and DIPEA (0.25 equiv, 0.18 mmol, 0.022 g) were added, and the mixture was stirred at room temperature for 5 min. After that time, a proper amine (1.2 equiv, 0.84 mmol) was added. The reaction mixture was stirred at 30 °C for 5 min in a microwave reactor operating at a frequency of 2.45 GHz and a radiating power of 200 W. Next, the reaction mixture was washed with distilled water and dried over sodium sulfate (VI). Then, the solvent was evaporated under reduced pressure. The crude mixture was purified by “flash” column chromatography over silica gel using dichloromethane/diethyl ether/methanol 75:20:5 (v/v/v) as an eluent affording a mixture of regioisomers (**9** and **10** or **11** and **12**). The regioisomers were further purified using a preparative HPLC system.

#### 4.2.12 2-(2-(4-Fluorophenyl)-6-methyl-1*H*-benzo[*d*]imidazole-1-yl)-1-(pyrrolidin-1-yl)ethan-1-one (**9**)

White solid, yield 4.06%, mp 194.1–195.3 °C. ^1^H NMR (500 MHz, CDCl_3_, 295 K): δ (ppm) 7.72 (t, *J* = 6.5 Hz, 2H), 7.68 (d, *J* = 8.0 Hz, 1H), 7.17 (t, *J* = 7.9 Hz, 2H), 7.11 (td, *J* = 1.0, 8.4 Hz, 1H), 7.03 (s, 1H), 4.79 (s, 2H), 3.58 (t, *J* = 6.9 Hz, 2H), 3.41 (t, *J* = 6.9 Hz, 2H), 2.48 (s, 3H), 2.04 (quin, *J* = 6.7 Hz, 2H), 1.92 (quin, *J* = 6.7 Hz, 2H). ^13^C NMR (126 MHz, CDCl_3_, 295 K): δ (ppm) 165.6, 163.5 (d, *J* = 249.3 Hz) carbons overlaps, 152.6, 140.5, 136.3, 133.3, 131.4 (d, *J* = 8.5 Hz), 125.1 (d, *J* = 3.6 Hz), 124.4, 119.3, 115.9 (d, *J* = 22.3 Hz), 109.5, 46.9, 46.4, 45.3, 26.2, 23.9, 21.8. ^19^F NMR (471 MHz CD_3_OD, 295 K): δ (ppm) −112.06 (s, 1F). LC–MS (ESI) calcd for C_20_H_20_FN_3_O, 338.162 [M + H^+^]; found, 338.299 [M + H^+^].

#### 4.2.13 2-(2-(4-Fluorophenyl)-5-methyl-1*H*-benzo[*d*]imidazole-1-yl)-1-(pyrrolidin-1-yl)ethan-1-one (**10**)

White solid, yield 7.9%, mp 208.7–210.20 °C. ^1^H NMR (500 MHz, CDCl_3_, 295 K): δ (ppm) = ppm 7.74–7.65 (m, 2 H), 7.46 (s, 1 H), 7.32–7.22 (m, 3 H), 7.12 (dd, *J* = 8.31, 0.86 Hz, 1 H), 4.98 (s, 2 H), 3.49 (t, *J* = 6.87 Hz, 2 H), 3.41 (t, *J* = 6.87 Hz, 2 H), 2.44 (s, 3 H), 2.01 (quin, J = 6.87 Hz, 2 H), 1.88 (quin, J = 6.87 Hz, 2 H). ^13^C NMR (126 MHz, CDCl_3_, 295 K): δ (ppm) 164.72, 163.81 (d, *J* = 249.5 Hz), 153.01, 142.71, 134.18, 132.58, 131.5 (d, *J* = 8.5 Hz), 126.10, 124.77, 119.63, 116.02 (d, *J* = 22.3 Hz), 109.16, aromatic carbons overlap, 47.07, 46.38, 45.90, 26.22, 23.99, 21.56. ^19^F NMR (471 MHz, CD_3_OD, 295 K): δ (ppm) −112.02 (s, 1 F). LC–MS (ESI) calcd for C_20_H_20_FN_3_O, 338.162 [M + H^+^]; found, 338.232 [M + H^+^].

#### 4.2.14 2-(2-(4-Fluorophenyl)-6-methyl-1*H*-benzo[*d*]imidazole-1-yl)-1-thiomorpholinoethan-1-one (**11**)

White solid, yield: 9.19%, mp 205.3–207.1 °C. ^1^H NMR (500 MHz, CDCl_3_, 294 K): δ (ppm) 7.71–7.62 (m, 3H), 7.18 (t, *J* = 8.6 Hz, 2H), 7.12 (d, *J* = 8.2 Hz, 1H), 6.99 (s, 1H), 4.83 (s, 2H), 3.96–3.89 (m, 2H), 3.74–3.67 (m, 2H), 2.67–2.62 (m, 2H), 2.57–2.52 (m, 2H), 2.48 (s, 3H). ^13^C NMR (126 MHz, CDCl_3_, 295 K): δ (ppm) 164.85, 163.41 (d, *J* = 249.3 Hz), 153.00, 143.03, 136.44, 134.28, 131.45 (d, *J* = 8.5 Hz), 126.18 (d, *J* = 3.6 Hz), 124.65, 119.93, 116.21 (d, *J* = 22.3 Hz), 109.47, aromatic carbons overlap, 47.86, 46.53, 45.21, 38.69, 27.98, 22.03. ^19^F NMR (471 MHz, CDCl_3_, 294 K): δ (ppm) −109.84 (s, 1F). LC–MS (ESI) calcd for C_20_H_20_FN_3_OS, 370.134 [M + H^+^]; found, 370.197 [M + H^+^].

#### 4.2.15 2-(2-(4-Fluorophenyl)-5-methyl-1*H*-benzo[*d*]imidazole-1-yl)-1-thiomorpholinoethan-1-one (**12**)

White powder, yield: 10.81%, mp 199.0–200.9 °C. ^1^H NMR (500 MHz, CDCl_3_, 295 K): δ (ppm) 7.68–7.65 (m, 2 H), 7.59 (s, 1 H), 7.19 (t, *J* = 7,7 Hz, 2 H), 7.11 (s, 2 H), 4.86 (s, 2 H), 3.92–3.90 (m, 2 H), 3.70–3.67 (m, 2 H), 2.64–2.61 (m, 2 H), 2.54–2.51 (m, 2 H), 2.48 (s, 3 H). ^13^C NMR (126 MHz, CDCl_3_, 295 K): δ (ppm) 164.75, 162.24 (d, *J* = 249.3 Hz), 152.59, 140.86, 136.44, 133.60, 131.38 (d, *J* = 8.5 Hz), 126.18 (d, *J* = 3.6 Hz), 124.65, 119.67, 116.03 (d, *J* = 22.3 Hz), 109.13, aromatic carbons overlap, 47.86, 46.44, 45.2, 38.69, 27.58, 21.67. ^19^F NMR (471 MHz, CDCl_3_, 295 K): δ (ppm) −109.84 (s, 1F). LC–MS (ESI) calcd for C_20_H_20_FN_3_OS, 370.134 [M + H^+^]; found, 370.263 [M + H^+^].

#### 4.2.16 General Procedure for the Preparation of Final Compounds (**13** and **14**)

The mixture of intermediates **7** and **8** (1 equiv, 0.70 mmol, 0.200 g) was solved in dry THF (4.29 mL/mmol) at 50 °C. Then, the reaction mixture was cooled down to 10 °C, and CDI (1.3 equiv, 0.91 mmol, 0.147 g) was added. After 2 h of activation at 10 °C, a proper amine (1.2 equiv, 0.84 mmol) was added. The reaction mixture was stirred at 40 °C for 12 h. After that time, the reaction mixture was quenched with water, and the aqueous phase was extracted with DCM. Next, the organic phase was washed with brine and dried over sodium sulfate (VI). Then, the solvent was evaporated under reduced pressure. The crude mixture was purified by “flash” column chromatography over silica gel using dichloromethane/diethyl ether/methanol 75:20:5 (v/v/v) as an eluent. The regioisomers were further purified using a preparative HPLC system.

#### 4.2.17 2-(2-(4-Fluorophenyl)-6-methyl-1*H*-benzo[*d*]imidazole-1-yl)-*N*-phenylacetamide (**13**)

White powder, yield: 21.2%, mp 202.5–205.0 °C. ^1^H NMR (500 MHz, CDCl_3_, 295 K): δ (ppm) 7.97 (br s, 1H), 7.68–7.61 (m, 3H), 7.49 (br d, *J* = 7.7 Hz, 2H), 7.31 (t, *J* = 7.9 Hz, 2H), 7.17–7.13 (m, 5H), 4.89 (s, 2H), 2.48 (s, 3H). ^13^C NMR (126 MHz, CDCl_3_, 295 K): δ (ppm) 165.07, 164.10 (d, *J* = 249.3 Hz), 153.05, 139.86, 137.90, 135.8, 134.15, 131.48 (d, *J* = 8.5 Hz), 128.68, 125.82, 124.46 (d, *J* = 3.6 Hz), 120.02, 119.84, 115.77 (d, *J* = 22.3 Hz), 109.87, aromatic carbons overlap, 37.54, 20.29. ^19^F NMR (471 MHz, CD_3_OD, 295 K): δ (ppm) −111.95 (s, 1 F). LC–MS (ESI) calcd for C_22_H_18_FN_3_O, 360.147 [M + H^+^]; found, 360.229 [M + H^+^].

#### 4.2.18 2-(2-(4-Fluorophenyl)-5-methyl-1*H*-benzo[*d*]imidazole-1-yl)-*N*-phenylacetamide (**14**)

White powder. yield: 2.8%, mp 207.3–209.2 °C. ^1^H NMR (500 MHz, CDCl_3_, 294 K): δ (ppm) 7.69–7.65 (m, 3H), 7.57 (s, 1H), 7.45 (d, *J* = 8.6 Hz, 2H), 7.31 (s, 2H), 7.20–7.15 (m, 5H), 4.90 (s, 2H), 2.49 (s, 3H). ^13^C NMR (126 MHz, CD_3_OD, 295 K): δ = 166.07, 164.10 (d, *J* = 249.3 Hz), 153.41, 147.12, 142.04, 139.86, 137.90, 136.25, 133.70, 131.55 (d, *J* = 8.5 Hz), 128.68, 125.81, 124.8, 124.46 (d, *J* = 3.6 Hz), 118.08, 115.77 (d, *J* = 22.3 Hz), 109.87, aromatic carbons overlap, 37.54, 20.55. ^19^F NMR (471 MHz, CD_3_OD, 295 K): δ (ppm) −111.88 (s, 1 F). LC–MS (ESI) calcd for C_22_H_18_FN_3_O, 360.147 [M + H^+^]; found, 360.229 [M + H^+^].

#### 4.2.19 General Procedure for the Preparation of Final Compounds (**15**, **16**, **18**, and **20–29**)

Intermediate **8** (1 equiv, 0.37 mmol, 0.100 g) was solved in dichloromethane (4.29 mL/mmol). Then, TBTU (2 equiv, 0.74 mmol, 0.238 g) and DIPEA (0.25 equiv, 0.09 mmol, 0.012 g) were added, and the mixture was stirred at room temperature for 5 min. After that time, a proper amine (1.2 equiv, 0.44 mmol) was added. The reaction mixture was stirred at 30 °C for 5 min in a microwave reactor with a frequency of 2.45 GHz and a radiating power of 200 W. Next, the reaction mixture was washed with distilled water and dried over sodium sulfate (VI). Then, the solvent was evaporated under reduced pressure. The crude mixture was purified by “flash” column chromatography over silica gel using dichloromethane/diethyl ether/methanol 70:29:1 (v/v/v) as an eluent.

#### 4.2.20 General Procedure for the Preparation of Final Compounds (**17**, **19**, **20**, and **21**)

Intermediate **8** (1 equiv, 0.37 mmol, 0.100 g) was solved in dry THF (4.29 mL/mmol) at 50 °C. Then, the reaction mixture was cooled down to 10 °C, and CDI (1.3 equiv, 0.48 mmol, 0.078 g) was added. After 2 h of activation at 10 °C, a proper amine (1.2 equiv, 0.44 mmol) was added. The reaction mixture was stirred at 40 °C for 12 h. After that time, the reaction mixture was quenched with water, and the aqueous phase was extracted with DCM. Next, the organic phase was washed with brine and dried over sodium sulfate (VI). Then, the solvent was evaporated under reduced pressure. The crude mixture was purified by “flash” column chromatography over silica gel using dichloromethane/diethyl ether/methanol 75:20:5 (v/v/v) as an eluent.

#### 4.2.21 2-(2-(4-Fluorophenyl)-1*H*-benzo[*d*]imidazole-1-yl)-*N*,*N*-dimethylacetamide (**15**)

White powder, yield: 19%, mp 182.3–183.1 °C. ^1^H NMR (500 MHz, DMSO-*d*_6_, 294 K): δ (ppm) 6.44–6.40 (m, 3H), 6.17–6.14 (m, 1H), 6.06–5.99 (m, 4H), 3.91–3.84 (m, 2H), 1.83 (s, 3H), 1.68 (s, 3H). ^13^C NMR (126 MHz, DMSO-*d*_6_, 294 K): δ (ppm) 166.0, 162.8 (d, *J* = 249.89 Hz), 152.1, 140.2, 134.8, 130.2 (d, *J* = 9.05 Hz), 125.1, 124.4 (d, *J* = 3.02 Hz), 124.2, 122.0, 121.6, 117.0, 114.5 (d, *J* = 22.33 Hz), 109.1, 44.5, 34.2, 33.7. ^19^F NMR (471 MHz, DMSO-*d*_6_, 294 K): δ (ppm) −113.03 (s, 1F). LC–MS (ESI) calcd for C_17_H_16_FN_3_O, 298.131 [M + H^+^]; found, 298.294 [M + H^+^].

#### 4.2.22 2-(2-(4-Fluorophenyl)-1*H*-benzo[*d*]imidazole-1-yl)-1-(pyrrolidin-1-yl)ethan-1-one (**16**)

White powder, yield: 65%, mp 212.9–214.0 °C. ^1^H NMR (500 MHz, CDCl_3_, 295 K): δ (ppm) 7.81–7.76 (m, 1H), 7.76–7.68 (m, 2H), 7.30–7.21 (m, 3H), 7.19–7.13 (m, 2H), 4.77 (s, 2H), 3.56–3.49 (m, 2H), 3.41–3.33 (m, 2H), 1.99 (quin, *J* = 6.8 Hz, 2H), 1.87 (quin, *J* = 6.9 Hz, 2H). ^13^C NMR (126 MHz, CDCl_3_, 295 K): δ (ppm) 164.69, 163.83 (d, *J* = 250.49 Hz), 153.33, 142.94, 136.33, 131.58, 131.51, 126.41, 126.39, 123.28, 122.80, 120.09, 116.03 (d, *J* = 2 1.73 Hz), 109.74, 47.03, 46.47, 46.01, 26.32, 24.10. ^19^F NMR (471 MHz, CDCl_3_, 295 K): δ (ppm) −110.25 (s, 1F). LC–MS (ESI) calcd for C_19_H_18_FN_3_O, 324.147 [M + H^+^]; found, 324.271 [M + H^+^].

#### 4.2.23 2-(2-(4-Fluorophenyl)-1*H*-benzo[*d*]imidazole-1-yl)-1-(piperazin-1-yl)ethan-1-one (**17**)

White powder, yield: 24%, mp 232.8–234.1 °C. ^1^H NMR (500 MHz, CD_3_OD, 294 K): δ (ppm) 7.68–7.65 (m, 3H), 7.42–7.40 (m, 1H), 7.30–7.27 (m, 4H), 5.14 (s, 2H), 3.53–3.48 (m, 4H), 2.80–2.75 (m, 4H). ^13^C NMR (126 MHz, DMSO-*d*_6_, 295 K): δ (ppm) 165.80, 163.04 (d, *J* = 247.47 Hz), 153.46, 141.76, 136.22, 132.43 (d, *J* = 9.05 Hz), 125.88 (d, *J* = 3.62 Hz), 123.20, 122.72, 118.41, 115.61 (d, *J* = 21.73 Hz), 110.05, aromatic carbons overlap, 45.58, 45.47, 45.28, 44.92, 42.77. ^19^F NMR (471 MHz, CD_3_OD, 294 K): δ (ppm) −111.83 (s, 1F). LC–MS (ESI) calcd for C_19_H_19_FN_4_O, 339.158 [M + H^+^]; found, 339.362 [M + H^+^].

#### 4.2.24 2-(2-(4-Fluorophenyl)-1*H*-benzo[*d*]imidazole-1-yl)-1-(4-methylpiperazin-1-yl)ethan-1-one (**18**)

White powder, yield: 86%, mp 172.3–173.1 °C. ^1^H NMR (500 MHz, CD_3_OD, 294 K): δ (ppm) 7.83–7.81 (m, 1H), 7.71–7.67 (m, 2H), 7.35–7.23 (m, 2H), 7.23–7.17 (m, 3H), 4.85 (s, 2H), 3.73–3.67 (m, 2H), 3.43 (t, 2H, *J* = 5.0 Hz), 2.48–2.45 (m, 2H), 2.42–2.37 (m, 2H), 2.37–2.35 (m, 3H). ^13^C NMR (126 MHz, CD_3_OD, 294 K): δ (ppm) 164.68, 162.82 (d, *J* = 250,49 Hz), 153.18, 142.90, 136.32, 131.50 (d, *J* = 9.05 Hz), 126.25 (d, *J* = 3.02 Hz), 123.37, 122.88, 120.14, 116.09 (d, *J* = 21.73 Hz), 109.66, aromatic carbons overlap, 55.07, 54.78, 46.14, 46.10, 45.01, 42.48. ^19^F NMR (471 MHz, CD_3_OD, 294 K): δ (ppm) −112.2 (s, 1F). LC–MS (ESI) calcd for C_20_H_21_FN_4_O, 353.173 [M + H^+^]; found, 353.384 [M + H^+^].

#### 4.2.25 1-(3,5-Dimethylpiperazin-1-yl)-2-(2-(4-fluorophenyl)-1*H*-benzo[*d*]imidazole-1-yl)ethan-1-one (**19**)

Yellow powder, yield: 55%, mp 109.6–112.4 °C. ^1^H NMR (500 MHz, DMSO-*d*_6_, 295 K): δ (ppm) 7.70–7.66 (m, 2H), 7.66–7.63 (m, 1H), 7.60 (s, 1H), 7.48–7.43 (m, 1H), 7.39–7.33 (m, 2H), 6.98 (s, 1H), 5.31–5.09 (m, 2H), 4.14–4.08 (m, 1H), 3.79–3.73 (m, 1H), 3.54–3.53 (m, 1H), 2.57–2.49 (m, 2H), 2.47–2.45 (m, 1H), 2.13–2.04 (m, 1H), 0.97–0.89 (m, 6H). ^13^C NMR (126 MHz, DMSO-*d*_6_, 295 K): δ (ppm) 165.29, 163.42 (d, *J* = 247.47 Hz), 153.13, 142.85, 137.19, 135.68, 131.77 (d, *J* = 9.05 Hz), 127.33 (d, *J* = 3.02 Hz), 122.95, 122.50, 119.51, 116.32 (d, *J* = 21.73 Hz), 111.28, 51.50 (d, *J* = 10.86 Hz), 50.90, 48.85, 46.37, 19.63, 19.40. ^19^F NMR (471 MHz, DMSO-*d*_6_, 295 K): δ (ppm) −111.83 (s, 1F). LC–MS (ESI) calcd for C_21_H_23_FN_4_O, 367.189 [M + H^+^]; found, 367.339 [M + H^+^].

#### 4.2.26 2-(2-(4-Fluorophenyl)-1*H*-benzo[*d*]imidazole-1-yl)-1-morpholinoethan-1-one (**20**)

White powder, yield: 28%, mp 232.7–232.9 °C. ^1^H NMR (500 MHz, CDCl_3_, 294 K): δ (ppm) 7.80–7.78 (m, 1H), 7.67–7.64 (m, 2H), 7.29–7.26 (m, 2H), 7.20–7.14 (m, 3H), 4.84 (s, 2H), 3.71–3.67 (m, 2H), 3.65–3.61 (m, 4H), 3.42–3.37 (m, 2H). ^13^C NMR (126 MHz, CDCl_3_, 294 K): δ (ppm) 164.96, 163.88 (d, *J* = 250.49 Hz), 153.19, 142.90, 136.24, 131.44 (d, *J* = 8.45 Hz), 126.17 (d, *J* = 3.02 Hz), 123.44, 122.98, 120.18, 116.14 (d, *J* = 22.33 Hz), 109.60, aromatic carbons overlap, 66.98, 66.48, 46.03, 45.47, 42.72. ^19^F NMR (471 MHz, CDCl_3_, 294 K): δ (ppm) −109.74 (s, 1F). LC–MS (ESI) calcd for C_19_H_18_FN_3_O_2_, 340.142 [M + H^+^]; found, 340.159 [M + H^+^].

#### 4.2.27 2-(2-(4-Fluorophenyl)-1*H*-benzo[*d*]imidazole-1-yl)-1-thiomorpholinoethan-1-one (**21**)

White powder, yield: 66%, mp 205.3–205.8 °C. ^1^H NMR (500 MHz, CDCl_3_, 294 K): δ (ppm) 7.81–7.76 (m, 1H), 7.67–7.61 (m, 2H), 7.30–7.24 (m, 2H), 7.21–7.14 (m, 3H), 4.84–4.80 (m, 2H), 3.91–3.86 (m, 2H), 3.67–3.62 (m, 2H), 2.63–2.59 (m, 2H), 2.54–2.49 (m, 2H). ^13^C NMR (126 MHz, CDCl_3_, 294 K): δ (ppm) 164.74, 162.90 (d, *J* = 249 Hz), 153.13, 142.89, 136.25, 131.44 (d, *J* = 8.45 Hz), 126.17 (d, *J* = 3.02 Hz), 123.45, 122.97, 120.19, 116.15 (d, *J* = 21.73 Hz), 109.61, aromatic carbons overlap, 47.86, 46.42, 45.22, 27.96, 27.58. ^19^F NMR (471 MHz, CDCl_3_, 294 K): δ (ppm) −109.63 (s, 1F). LC–MS (ESI) calcd for C_19_H_18_FN_3_OS, 356.119 [M + H^+^]; found, 356.175 [M + H^+^].

#### 4.2.28 2-(2-(4-Fluorophenyl)-1*H*-benzo[*d*]imidazole-1-yl)-*N*-phenylacetamide (**22**)

White powder, yield: 45%, mp 236.7–239.0 °C. ^1^H NMR (500 MHz, CD_3_OD, 294 K): δ (ppm) 7.78 (dd, *J* = 5.2, 8.9 Hz, 2H), 7.71–7.67 (m, 1H), 7.54–7.49 (m, 2H), 7.46–7.43 (m, 1H), 7.33–7.31 (m, 1H), 7.30–7.29 (m, 1H), 7.28–7.26 (m, 2H), 7.26–7.23 (m, 2H), 7.11–7.06 (m, 1H), 5.02 (s, 2H). ^13^C NMR (126 MHz, CD_3_OD, 294 K): δ (ppm) 165.91, 164.10 (d, *J* = 249.89 Hz), 153.44, 141.79, 137.79, 135.97, 131.60 (d, *J* = 8.45 Hz), 128.71, 125.65 (d, *J* = 3.02 Hz), 124.45, 123.44, 122.97, 119.91, 118.64, 115.70 (d, *J* = 22.33 Hz), 110.07, aromatic carbons overlap, 48.70. ^19^F NMR (471 MHz, CD_3_OD, 294 K): δ (ppm) −113.2 (s, 1F). LC–MS (ESI) calcd for C_21_H_16_FN_3_O, 346.131 [M + H^+^]; found, 346.362 [M + H^+^].

#### 4.2.29 3-(2-(2-(4-Fluorophenyl)-1*H*-benzo[*d*]imidazole-1-yl)acetamido)benzamide (**23**)

Orange powder, yield: 38%, mp 181.2–182.3 °C. ^1^H NMR (500 MHz, DMSO-*d*_6_, 295 K): δ (ppm) 10.63 (s, 1H), 8.03–8.02 (m, 1H), 7.92 (s, 1H), 7.84–7.81 (m, 2H), 7.71–7.67 (m, 2H), 7.56–7.52 (m, 2H), 7.40–7.34 (m, 3H), 7.35–7.31 (m, 1H), 7.27–7.23 (m, 2H), 5.11 (s, 2H), 3.13 (s, 1H). ^13^C NMR (126 MHz, DMSO-*d*_6_, 295 K): δ (ppm) 168.18, 166.34, 163.51 (d, *J* = 247.47 Hz), 153.28, 142.90, 138.96, 136.91, 135.72, 132.07 (d, *J* = 9.05 Hz), 129.28, 127.12 (d, *J* = 3.02 Hz), 123.26, 123.05, 122.79, 122.51, 119.68, 119.41, 116.41 (d, *J* = 21.73 Hz), 111.08, 48.16. ^19^F NMR (471 MHz, DMSO-*d*_6_, 295 K): δ (ppm) −110.94 (s, 1F). LC–MS (ESI) calcd for C_22_H_17_FN_4_O_2_, 389.142 [M + H^+^]; found, 389.203 [M + H^+^].

#### 4.2.30 2-(2-(4-Fluorophenyl)-1*H*-benzo[*d*]imidazole-1-yl)-*N*-(2-methoxyphenyl)acetamide (**24**)

White powder, yield: 25%, mp 206.9–207.2 °C. ^1^H NMR (500 MHz, CDCl_3_, 295 K): δ (ppm) 8.27 (dd, *J* = 1.7, 8.0 Hz, 1H), 7.97 (s, 1H), 7.89–7.87 (m, 1H), 7.76–7.73 (m, 2H), 7.42–7.35 (m, 3H), 7.22–7.19 (m, 2H), 7.05 (td, *J* = 1.4, 8.0 Hz, 1H), 6.95 (td, *J* = 1.4, 8.0 Hz, 1H), 6.80 (dd, *J* = 1.3, 8.2 Hz, 1H), 4.96 (s, 2H), 3.54 (s, 3H). ^13^C NMR (126 MHz, CDCl_3_, 295 K): δ (ppm) 164.50, 163.04 (d, *J* = 250,49 Hz), 153.25, 148.20, 143.16, 135.69, 131.47 (d, *J* = 9.05 Hz), 126.28, 125.55 (d, *J* = 3.62 Hz), 125.05, 123.95, 123.60, 121.15, 120.40, 120.23, 116.57 (d, *J* = 21.73 Hz), 110.21, 109.91 (aromatic carbons overlap), 55.66, 49.18. ^19^F NMR (471 MHz, CDCl_3_, 295 K): δ (ppm) −109.27 (s, 1F). LC–MS (ESI) calcd for C_22_H_18_FN_3_O_2_, 376.142 [M + H^+^]; found, 376.111 [M + H^+^].

#### 4.2.31 2-(2-(4-Fluorophenyl)-1*H*-benzo[*d*]imidazole-1-yl)-*N*-(3-methoxyphenyl)acetamide (**25**)

White powder, yield: 71%, mp 149.1–149.9 °C. ^1^H NMR (500 MHz, CDCl_3_, 294 K): δ (ppm) 8.75 (s, 1H), 7.62–7.59 (m, 3H), 7.26 (dd, *J* = 1.3, 5.3 Hz, 2H), 7.24–7.15 (m, 3H), 7.10–7.07 (m, 2H), 7.03 (dd, *J* = 1.1, 8.0 Hz, 1H), 6.70–6.67 (m, 1H), 4.78 (s, 2H), 3.72 (s, 3H). ^13^C NMR (126 MHz, CDCl_3_, 294 K): δ (ppm) 165.25, 163.97 (d, *J* = 251.70 Hz), 160.26, 153.35, 142.71,138.27, 135.76, 131.34 (d, *J* = 8.45 Hz), 129.93, 125.24 (d, *J* = 3.62 Hz), 123.99, 123.75, 119.85, 116.38 (d, *J* = 21.73 Hz), 112.63, 111.03, 109.60, 106.41, 55.41, 48.79. ^19^F NMR (471 MHz, CDCl_3_, 294 K): δ (ppm) −108.96 (s, 1F). LC–MS (ESI) calcd for C_22_H_18_FN_3_O_2_, 376.142 [M + H^+^]; found, 376.244 [M + H^+^].

#### 4.2.32 2-(2-(4-Fluorophenyl)-1*H*-benzo[*d*]imidazole-1-yl)-*N*-(4-methoxyphenyl)acetamide (**26**)

Beige powder, yield: 41%, mp 255.1–255.7 °C. ^1^H NMR (500 MHz, DMSO-*d*_6_, 295 K): δ (ppm) 10.35 (s, 1H), 7.84–7.81 (m, 2H), 7.69–7.67 (m, 1H), 7.53–7.51 (m, 1H), 7.47–7.44 (m, 2H), 7.40–7.36 (m, 2H), 7.28–7.22 (m, 2H), 6.88–6.84 (m, 2H), 5.06 (s, 2H), 3.32 (s, 3H). ^13^C NMR (126 MHz, DMSO-*d*_6_, 295 K): δ (ppm) 165.63, 163.51 (d, *J* = 247.47 Hz), 156.10, 153.28, 142.90, 136.93, 132.08 (d, *J* = 8.45 Hz), 127.17 (d, *J* = 3.02 Hz), 123.22, 122.75, 121,65, 121.41, 119.67, 116.37 (d, *J* = 21.73 Hz), 114.50, 111.07, aromatic carbons overlap, 55.71, 48.06. ^19^F NMR (471 MHz, DMSO-*d*_6_, 295 K): δ (ppm) −111.00 (s, 1F). LC–MS (ESI) calcd for C_22_H_18_FN_3_O_2_, 376.142 [M + H^+^]; found, 376.178 [M + H^+^].

#### 4.2.33 *N*-(2-Fluorophenyl)-2-(2-(4-fluorophenyl)-1*H*-benzo[*d*]imidazole-1-yl)acetamide (**27**)

White powder, yield: 30%, mp 212.1–212.8 °C. ^1^H NMR (500 MHz, CDCl_3_, 295 K): δ (ppm) 8.11 (dt, *J* = 2.4, 7.5 Hz, 1H), 7.95 (br s, 1H), 7.83–7.79 (m, 1H), 7.73–7.66 (m, 2H), 7.38–7.31 (m, 3H), 7.21–7.17 (m, 2H), 7.14–7.08 (m, 2H), 7.06–7.02 (m, 1H), 4.94 (s, 2H). ^13^C NMR (126 MHz, CDCl_3_, 295 K): δ (ppm) 165.15, 164.01 (d, *J* = 251.70 Hz), 153.27, 153.19 (d, *J* = 245.06 Hz), 142.90, 135.68, 131.44 (d, *J* = 8.45 Hz), 126.11 (d, *J* = 7.85 Hz), 125.35, 124.90 (d, *J* = 10.87), 124.76 (d, *J* = 3.02 Hz), 124.12, 123.77, 122.99, 120.35, 116.45 (d, *J* = 21.73 Hz), 115.31 (d, *J* = 18.71 Hz), 109.60, aromatic carbons overlap, 48.82. ^19^F NMR (471 MHz, CDCl_3_, 295 K): δ (ppm) −116.60 (s, 1F), −109.09 (s, 1F). LC–MS (ESI) calcd for C_21_H_15_F_2_N_3_O, 364.122 [M + H^+^]; found, 364.282 [M + H^+^].

#### 4.2.34 *N*-(3-Fluorophenyl)-2-(2-(4-fluorophenyl)-1*H*-benzo[*d*]imidazole-1-yl)acetamide (**28**)

White powder, yield: 15%, mp 182.3–182.7 °C. ^1^H NMR (500 MHz, CDCl_3_, 294 K): δ (ppm) 8.91 (s, 1H), 7.67 (br dd, *J* = 5.2, 8.9 Hz, 3H), 7.53–7.49 (m, 1H), 7.33–7.28 (m, 3H), 7.23–7.19 (m, 2H), 7.16–7.12 (t, *J* = 8.2 Hz 2H), 6.85–6.81 (m, 1H), 4.89 (s, 2H).^13^C NMR (126 MHz, CDCl_3_, 294 K): δ (ppm) 165.33, 163.51 (d, *J* = 248.68 Hz), 163.46 (d, *J* = 245.06 Hz), 153.36, 142.63, 138.71 (d, *J* = 10.26), 135.76, 131.43 (d, *J* = 8.45 Hz), 130.30 (d, *J* = 9.05 Hz), 125.25 (d, *J* = 3.02 Hz), 124.06, 123.77, 119.99, 116.40 (d, *J* = 22.33 Hz), 115.67 (d, *J* = 3.02 Hz), 112.02 (d, *J* = 21.73 Hz), 109.63, 107.96 (d, *J* = 25.95 Hz), aromatic carbons overlap, 48.75. ^19^F NMR (471 MHz, CDCl_3_, 294 K): δ (ppm) −110.85 (s, 1F), −108.98 (s, 1F). LC–MS (ESI) calcd for C_21_H_15_F_2_N_3_O, 364.122 [M + H^+^]; found, 364.216 [M + H^+^].

#### 4.2.35 *N*-(4-Fluorophenyl)-2-(2-(4-fluorophenyl)-1*H*-benzo[*d*]imidazole-1-yl)acetamide (**29**)

White powder, yield: 77%, mp 248.0–248.2 °C. ^1^H NMR (500 MHz, CDCl_3_, 295 K): δ (ppm) 9.12 (s, 1H), 7.64–7.60 (m, 3H), 7.52–7.49 (m, 2H), 7.29–7.22 (m, 3H), 7.08 (s, 2H), 6.99–6.95 (m, 2H), 4.82 (s, 2H). ^13^C NMR (126 MHz, CDCl_3_, 295 K) = 165.37, 163.96 (d, *J* = 252.30 Hz), 159.86 (d, *J* = 245.06 Hz), 153.36, 142.70, 135.81, 133.29, 131.38 (d, *J* = 8.45 Hz), 125.35 (d, *J* = 3.62 Hz), 123.91, 123.65, 122.47 (d, *J* = 7.85 Hz), 119.84, 116.31 (d, *J* = 21.73 Hz), 115.84 (d, *J* = 22.33 Hz), 109.62, aromatic carbons overlap, 48.57. ^19^F NMR (471 MHz, CDCl_3_, 295 K): δ (ppm) −109.09 (s, 1F), −116.60 (s, 1F). LC–MS (ESI) calcd for C_21_H_15_F_2_N_3_O, 364.122 [M + H^+^]; found, 364.068 [M + H^+^].

### 4.3 Radioligand Binding Assay for the GABA-A Receptor

Rat brains were homogenized in 20 volumes of ice-cold 50 mM Tris-HCl buffer (pH 7.4) with the use of an ULTRA TURAX homogenizer. The homogenate was subsequently centrifuged at 20,000*g* for 20 min (0–4 °C). The obtained supernatant was separated, and the pellet was rehomogenized in 20 volumes of ice-cold Tris-HCl buffer (pH 7.4) 50 mM. The acquired homogenate was again centrifuged as described above. The pellet was resuspended and further centrifuged three times. The final pellet was stored at −80 °C for a minimum period of 18 h. On the day of the study, the pellet was defrosted at room temperature, resuspended in 20 volumes of ice-cold 50 mM Tris-HCl buffer (pH 7.4), and centrifuged at 20,000*g* for 25 min (0–4 °C). To determine specific binding to GABA-A receptors, [^3^H]-flunitrazepam (spec. act. 80 Ci/mmol, ARC) was used. Then, 150 μL of the tissue suspension, 50 μL of 10 μM diazepam (displacer), 50 μL of 1 nM [^3^H]flunitrazepam, and 50 μL of the analyzed compound were incubated at 4 °C for 20 min. The concentrations of the compounds analyzed ranged from 1.0 × 10^–10^ to 1.0 × 10^–5^ M. The incubation was quenched by rapid filtration over glass fiber filters FilterMate B (PerkinElmer) using a 96-well FilterMate harvester (PerkinElmer). Five rapid washes were performed with ice-cold 50 mM Tris-HCl buffer, pH 7.4. The filter mat was dried in a microwave, placed in a plastic bag (PerkinElmer), and soaked with 10 mL of liquid scintillation cocktail Ultima Gold MV (PerkinElmer), and the filter bag was sealed. The radioactivity of the filter was measured on a MicroBeta TriLux 1450 scintillation counter (PerkinElmer). Radioligand binding data were analyzed using iterative curve fitting routines of GraphPad Prism 5.0 software (GraphPad, Inc., La Jolla, CA) and using the three built-in parameter logistic model that describes ligand competition binding to radioligand-labeled sites. The log IC_50_ (i.e., the log of the ligand concentration that reduces a specific radioligand binding by 50%) estimated from the data is used to obtain the *K*_*i*_ values by applying the Cheng–Prusoff approximation.

### 4.4 Electrophysiological Studies

Electrophysiology studies were performed using the QPatch16X automatic patch clamp platform (Sophion Biosciences) and HEK293 cells, stably expressing the α_1_β_2_γ_2_ subunits of the human GABA-A receptor. Prior to the assay recordings, the cells were transferred from the culture flask through a TrypLE Express solution (LifeTechnologies) and resuspended in serum-free media. The cells were placed in the lidless microtube, located onboard the automated electrophysiology instrument. The cells were then automatically relocated to a built-in centrifuge, spun down, and washed in Ringer’s extracellular solution. The cells were transferred to the pipetting wells of a single-use 16-channel planar patch chip plate (QPlate 16X), and gigaseals were formed upon implementation of a combined suction/voltage protocol. Subsequent suction induced whole-cell configuration. The chloride currents passed via the GABA-A receptor were recorded for 7 s after single addition of a tested molecule. Through whole-cell recording, the holding potential was set to −90 mV. The assay was performed at room temperature. The extracellular solution was composed of the following ingredients: consisted of 2 mM 4KCl, 145 mM NaCl, 10 mM HEPES, CaCl_2_, 1 mM MgCl_2_, 10 mM glucose (pH 7.4, 300 mOsm), and intracellular solution contained 140 mM CsF, 1 mM EGTA, 5 mM CsOH, 10 mM HEPES, and 20 mMNaCl (pH 7.2, 320 mOsm). The screening assays were established in the instrument software by sequential application of 10 μM GABA (reference agonist AG1), 1 μM examined molecule compounds, and compounds co-administered with 10 μM GABA (T2); second addition of 10 μM GABA (AG2); 10 μM bicuculline (reference antagonist) along with 10 μM GABA (ATG). The intermission among the addition of particular molecules was 60 s. In a typical procedure, 5 μL of ligand was added to the cells, followed by 3 s of washout with an extracellular solution (two times 5 mL). For the allosteric modulator/antagonist mode, the cells were preincubated with the tested compound alone for 50 s, followed by the addition of the reference agonist. The data obtained were processed using QPatch Assay Software (v5.0, Sophion Biosciences) and are represented as the mean of at least three independent experiments performed on separate cells. The validation criteria for a single test were current amplitude evoked by adding GABA greater than 500 pA and a change between cells’ response to GABA applications (AG1 and AG2) not greater than 25%. The relative compound efficiency was analyzed as the baseline-corrected ratio of maximal current amplitudes induced by the addition of tested compounds and reference agonist (T1-ATG/AG1-ATG or T2-ATG/AG1-ATG). The obtained raw current recordings were normalized and represented as % of the current amplitude induced by a reference agonist (GABA = 100%) using the QPatch Assay Software (v5.0, Sophion Biosciences).^24^

### 4.5 Solubility in pH = 7.4

Quantitative HPLC analyses were performed on a Waters Alliance e2695 Separations Module (Waters, Milford, CT, USA) with a detector 2998 Photodiode Array (PDA) detector (Waters, Milford, CT, USA) and the Chromolithe SpeedROD RP-18e 50–4.6 mm column (Merck, KGaA, Darmstadt, Germany). The column was kept at 30 °C. The conditions applied are as follows: eluent A (water/0.1% formic acid), eluent B (MeCN/0.1% formic acid), a flow rate of ,5 mL/min, a gradient of 0–100% B over 3 min, and an injection volume of 10 μL. Each sample was injected in triplicate. The spectra were analyzed at 255 (perphenazine) and 291 nm (9, 23). Standard solutions of the tested compounds and the reference compound were prepared in methanol at a concentration of 1 mg/mL. The standard solutions were diluted with methanol to obtain several solutions with a concentration of 0.5–0.125 μg/mL, which were used to prepare calibration curves (AUC vs concentration μg/mL). Next, 2 mg of the compound tested was dissolved in 1 mL of Dulbecco’s phosphate-buffered saline (DPBS), and the mixture was constantly agitated at 20 °C for 24 h in a thermoshaker. After that time, the mixtures were filtered through a cellulose acetate syringe filter (pore size 0.22 μm) and transferred to a chromatographic vial and analyzed using HPLC. For quantification, areas under the peaks of the investigated compounds on DAD chromatograms were used. Solubility was determined using the calibration curves.

### 4.6 Metabolic Stability Assay

The in vitro evaluation of the metabolic stability of the selected compound was performed using human liver microsomes (HLMs) (Sigma-Aldrich, St. Louis, MO, USA) as described previously.^25^ The reaction mixture contained 50 μM of tested molecules HLMs (1 mg/mL) in 10 mM tris–HCl buffer. Following 5 min of preincubation, 50 μL of the NADPH Regeneration System (Promega, Madison, WI, USA) was added to induce the reaction. Next, the resulting reaction mixture was incubated at 37 °C for 120 min. The reaction was quenched with the addition of 200 μL of cold extra pure methanol and then centrifuged at 14,000 rpm for 15 min. The resulting supernatant was analyzed using an LC/MS Waters ACQUITY TQD system (Waters, Milford, USA).

### 4.7 Hepatotoxicity Assay

The human hepatocellular carcinoma cells (HepG2) were cultured using standard procedures (protocol from ATCC). The cells were cultured in Dulbecco’s Modified Eagle’s Medium—high glucose (DMEM, Thermo Fisher) supplemented with 10% fetal bovine serum (Thermo Fisher) with added 100 IU/mL penicillin (Merck) and 100 μg/mL streptomycin (Merck). The cells were cultured in flasks with an area of 175 cm^2^ (Nunc) and incubated at 37 °C, 5% CO_2_. For the test of compounds with the HepG2 cells line, hepatocytes were seeded on a 96-well culture plate (Falcon) at a density of 2 × 10^4^ cells per well in a fresh medium. The cells were grown for 24 h in the incubator (37 °C, 5% CO_2_) before performing experiments. After dilution, tested compounds were added and incubated for 24 h in aseptic conditions. Stock solutions were prepared in the concentration of 10^–2^ M for test and reference compounds. A minimum of 1 mg of each tested compound was weighed and dissolved in an appropriate volume of dimethyl sulfoxide. Serial dilutions were prepared in DMSO, and then the diluted compounds were transferred to PBS, mixed, and put to medium with adherent cells. Before assays, eventual precipitation or opalescence was checked. All experiments were performed in triplicate, in three independent experiments.

**Cell viability** was measured using the PrestoBlue reagent (Thermo Fisher). The PrestoBlue reagent is a resazurin-based solution that functions as a cell viability indicator is used. Metabolically active cells are capable of reducing the PrestoBlue reagent, with the colorimetric changes used as an indicator to quantify the viability of cells in culture. This change can be determined by measuring the fluorescence. After 24 h of incubation with the compounds, the PrestoBlue reagent was added to the wells of a microplate in an amount equals to one-tenth of the remaining medium volume. After 30 min of incubation at 37 °C, the fluorescence intensity (EX 530; EM 580 nm) was measured by using a multifunction plate reader (POLARstar Omega, BMG Labtech, Germany). Viability values were calculated as a percentage of live cells with respect to the control sample. **Cell membrane damage** was measured using the bioluminescent ToxiLight bioassay (Lonza). It quantitatively measures the release of adenylate kinase (AK) from the membranes of damaged cells. AK is a protein presented in all eukaryotic cells, which is released into the culture medium when cells die. The enzyme actively phosphorylates ADP, and the resultant ATP is then measured using the bioluminescent firefly luciferase reaction with the ToxiLight reagent. The emitted light intensity expressed as a RLU value is linearly related to the adenylate kinase activity. After 24 h of treatments, 5 μL of the clear fluid above a sediment was transferred into a 384-well plate (PerkinElmer). Then, 20 μL of the Adenylate Kinase Detection Reagent (AKDR) was added. The luminescence was measured after 10 min of incubation with a plate reader POLARstar Omega (BMG Labtech). The results are expressed as a percentage of control, which corresponds to the percentage of dead cells. The loss of **mitochondrial membrane potential** was assessed using a lipophilic cationic fluorescent probe, JC-1 (Thermo Fisher). The cells were incubated with the test compounds for 24 h at 37 °C in 5% CO_2_. Subsequently, the cells were incubated with 2 μg/mL JC-1 for 40 min at 37 °C. After incubation, the fluorescence intensity was measured at EX 530 nm and EM 580 nm for aggregates and EX 490 nm and EM 530 nm for monomers using a POLARstar Omega microplate reader (BMG Labtech, Germany). The results were calculated as the ratio of the fluorescence of aggregates to monomers. The results are expressed as a percentage of control. Statistical analysis was performed using the program GraphPad. All values are expressed as mean with SD. Differences among groups were evaluated by one-way ANOVA followed by post-hoc analysis (Dunnett’s multiple comparisons tests) and were considered statistically significant if *p* < 0.05.

## Supporting Information Available

^1^H NMR, ^13^C NMR, COSY, and LCMS/MS spectra of the selected final compounds. LCMS/MS spectra of the tested compounds following the metabolic stability assay (PDF)
