# Supplementary material for: 2-(4-Fluorophenyl)-1H-benzo[d]imidazole as a Promising Template for the Development of Metabolically Robust, α1β2γ2GABA-A Receptor-Positive Allosteric Modulators
Source: ACS Chem Neurosci. 2023 Feb 27;14(6):1166–80. doi: 10.1021/acschemneuro.2c00800 (PMC10020958; doi:10.1021/acschemneuro.2c00800)
Supplement: Supplementary file 1 — cn2c00800_si_001.pdf [file cn2c00800_si_001.pdf]

Supporting Information

The 2-(4-fluorophenyl)-1*H*-benzo[*d*]imidazole as a promising template for the development of metabolically robust, α1β2γ2GABA-A receptor positive allosteric modulators

Monika Marcinkowska[*a], Nikola Fajkis-Zajączkowska[a], Katarzyna Szafrańska[a], Jakub Jończyk[a], Agata Siwek[b], Barbara Mordyl[b], Tadeusz Karcz[c], Gniewomir Latacz[c], Marcin Kolaczkowski[a]

[a]Department of Medicinal Chemistry, Faculty of Pharmacy, Jagiellonian University Medical College, 9 Medyczna St., 30-688 Kraków, Poland

[b]Department of Pharmacobiology, Faculty of Pharmacy Jagiellonian University Medical College, 9 Medyczna St., 30-688 Kraków, Poland

[c]Department of Technology and Biotechnology of Drugs, Faculty of Pharmacy, Jagiellonian University Medical College, 9 Medyczna St., 30-688 Kraków, Poland

*Corresponding Author Information:

Phone: (+48)126205460

E-mail: monika.marcinkowska@uj.edu.pl

1. NMR and UPLC-UV-MS spectra of selected final compounds.

3-(2-(2-(4-fluorophenyl)-1H-benzo[d]imidazol-1-yl)acetamido)benzamide (23)

LC-MS (ESI) calcd for $C_{22}H_{17}FN_4O_2$ 389.12 [M + H$^+$], found 389.203 [M + H$^+$].

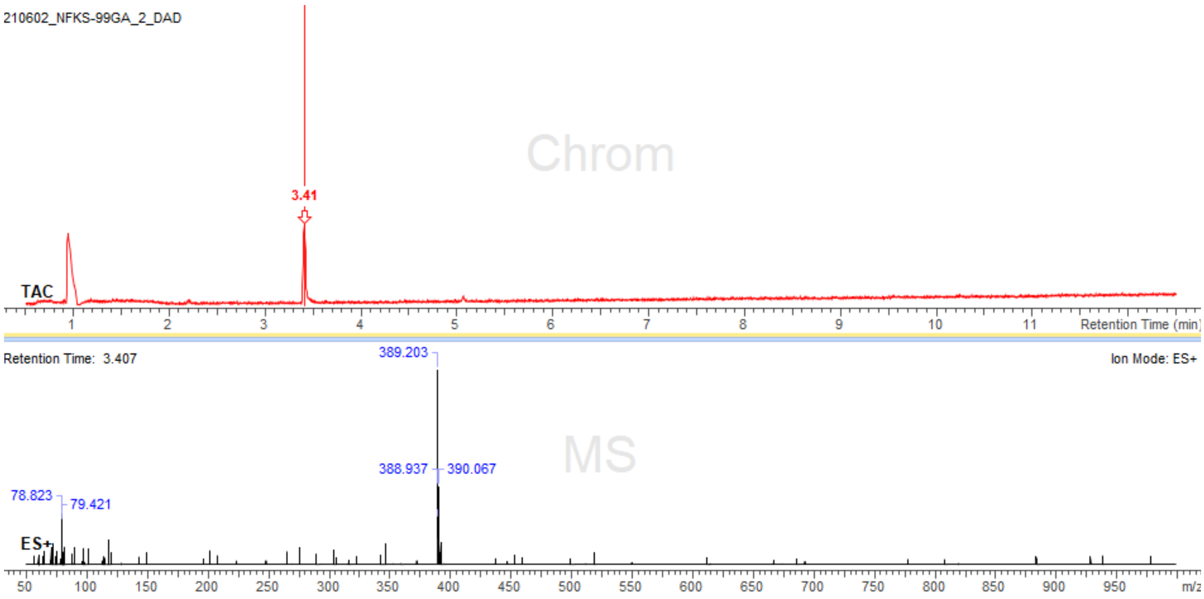

^{1}H NMR (500 MHz, DMSO-d_6)

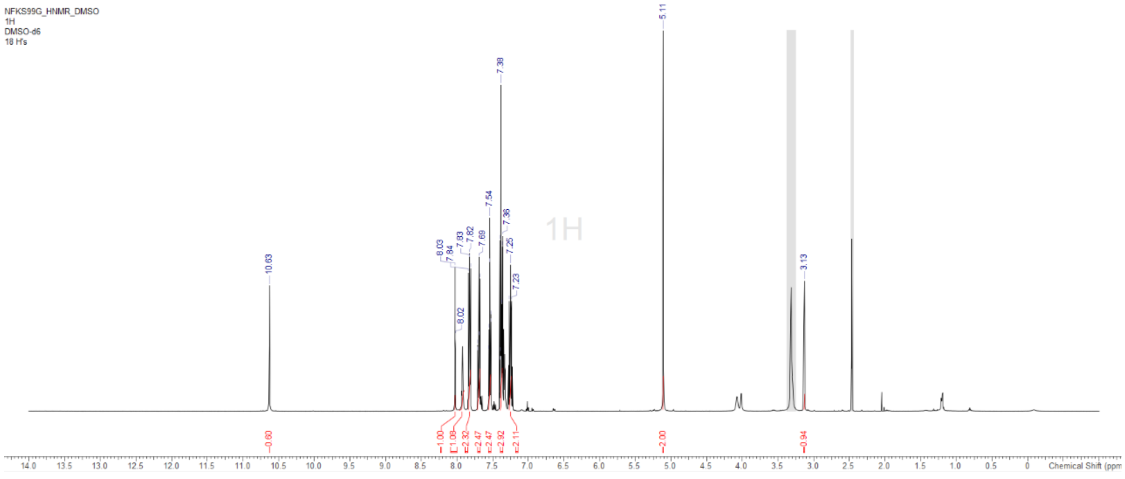

^{13}C NMR (126 MHz, DMSO-d$_6$)

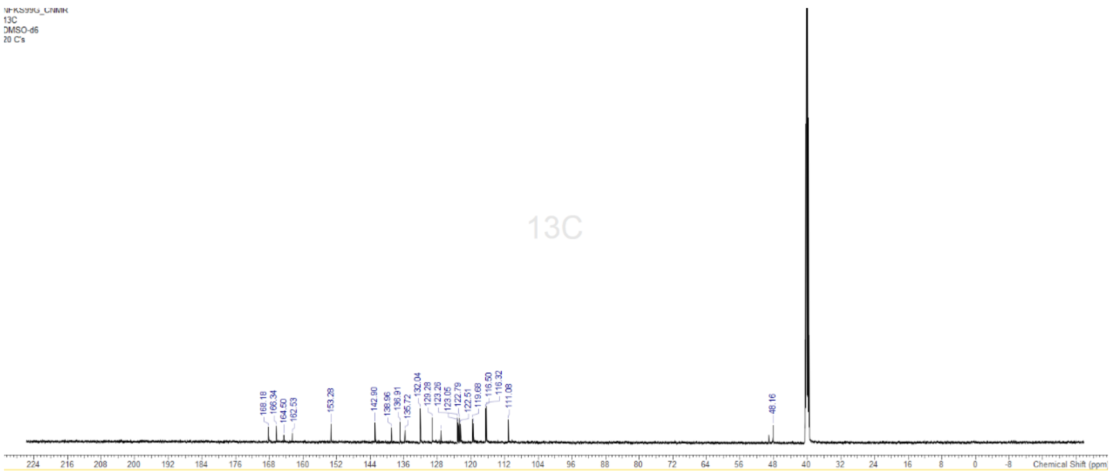

2-(2-(4-fluorophenyl)-1*H*-benzo[*d*]imidazol-1-yl)-1-(pyrrolidin-1-yl)ethan-1-one (16)

LC-MS (ESI) calcd for $C_{19}H_{18}FN_3O$ 324.12 [M + H$^+$], found 324.271 [M + H$^+$].

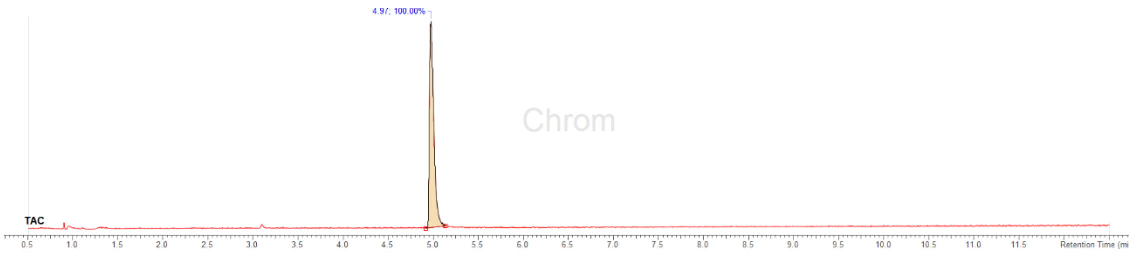

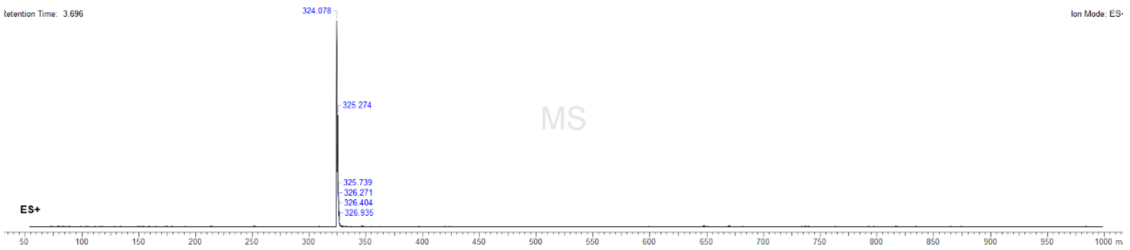

^{1}**H NMR** (500 MHz, $CDCl_3$)

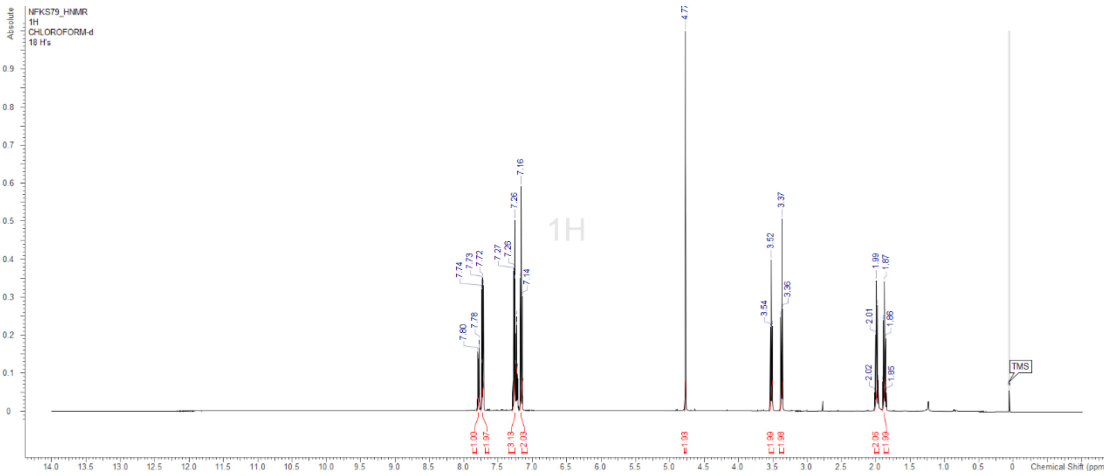

^{13}**C NMR** (126 MHz, $CDCl_3$)

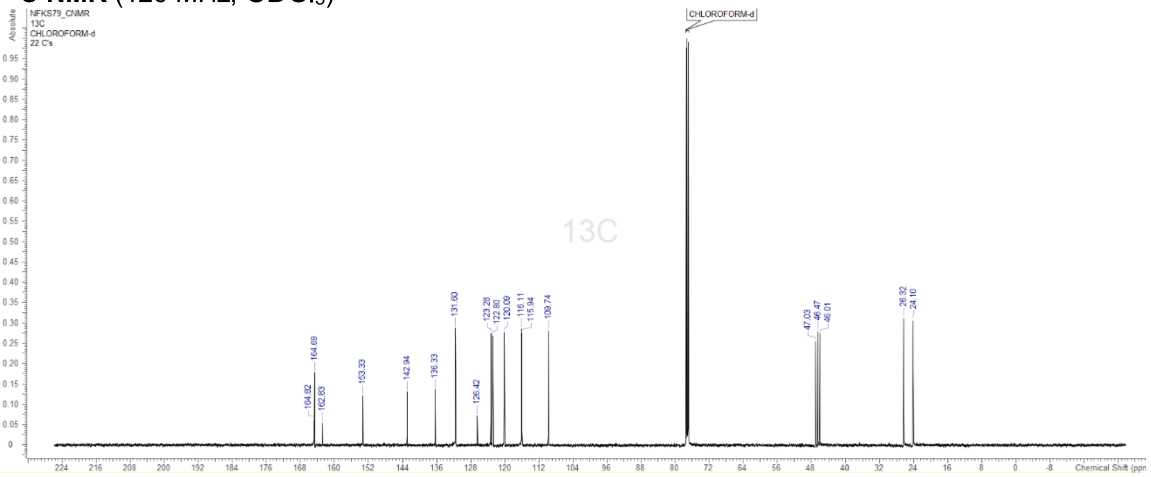

2-(2-(4-fluorophenyl)-6-methyl-1*H*-benzo[*d*]imidazol-1-yl)-1-(pyrrolidin-1-yl)ethan-1-one (9)

LC-MS (ESI) calcd for $C_{20}H_{20}FN_3O$: 338.16 [M + H$^+$], found: 338.299 [M + H$^+$].

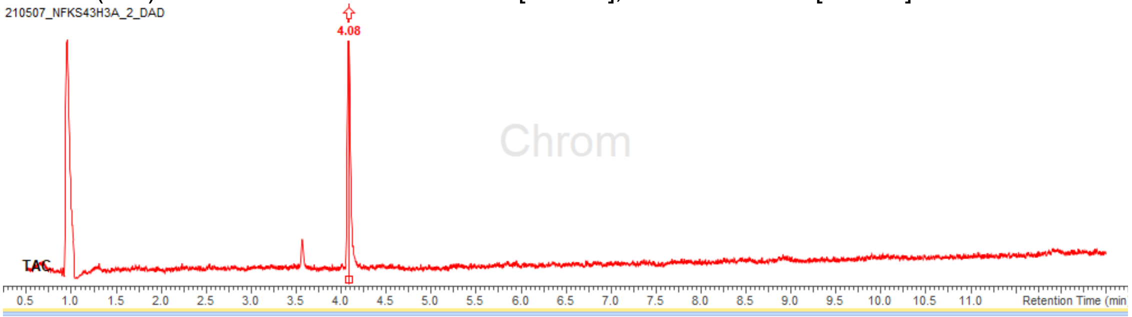

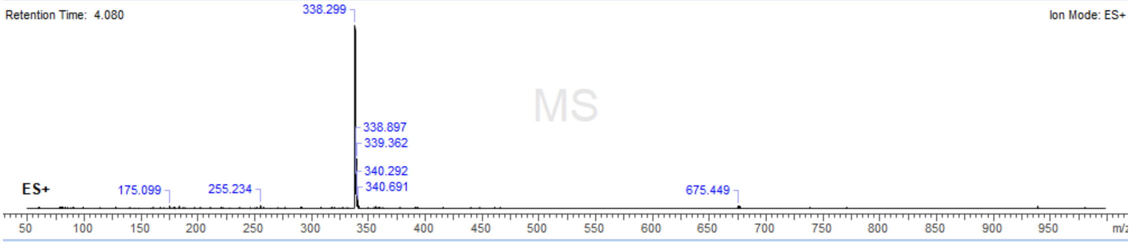

[1]**HNMR** (500 MHz, $CDCl_3$)

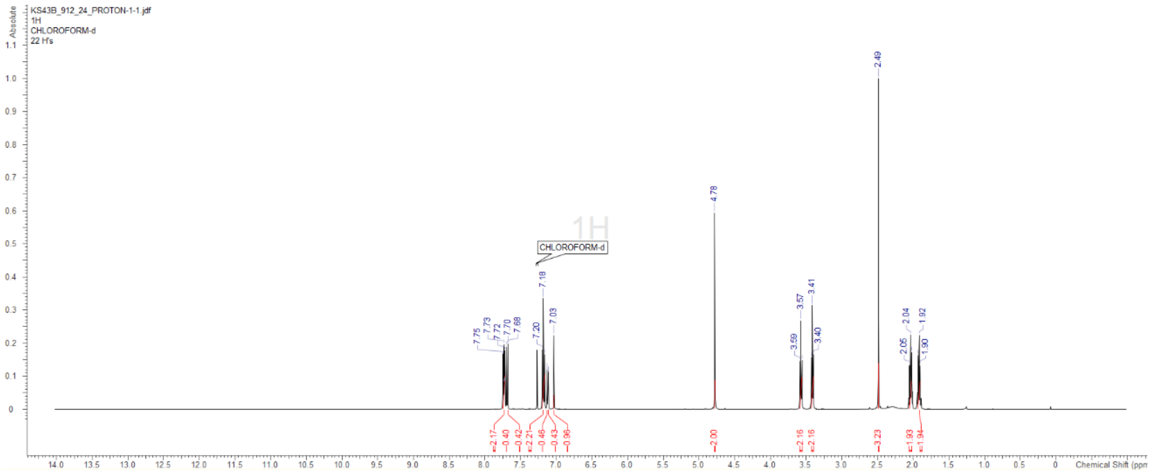

[13]**C NMR** (126 MHz, $CDCl_3$)

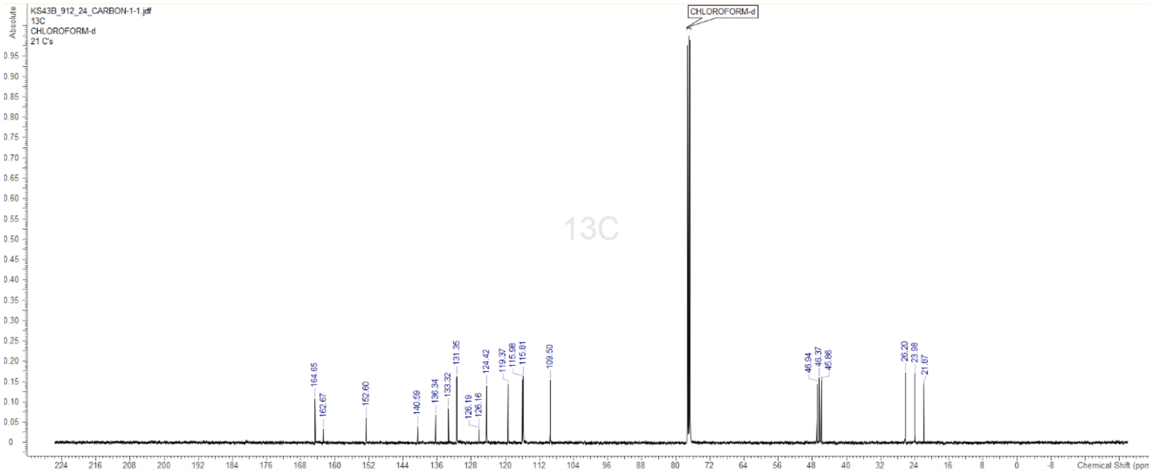

COSY (500 MHz, $CDCl_3$)

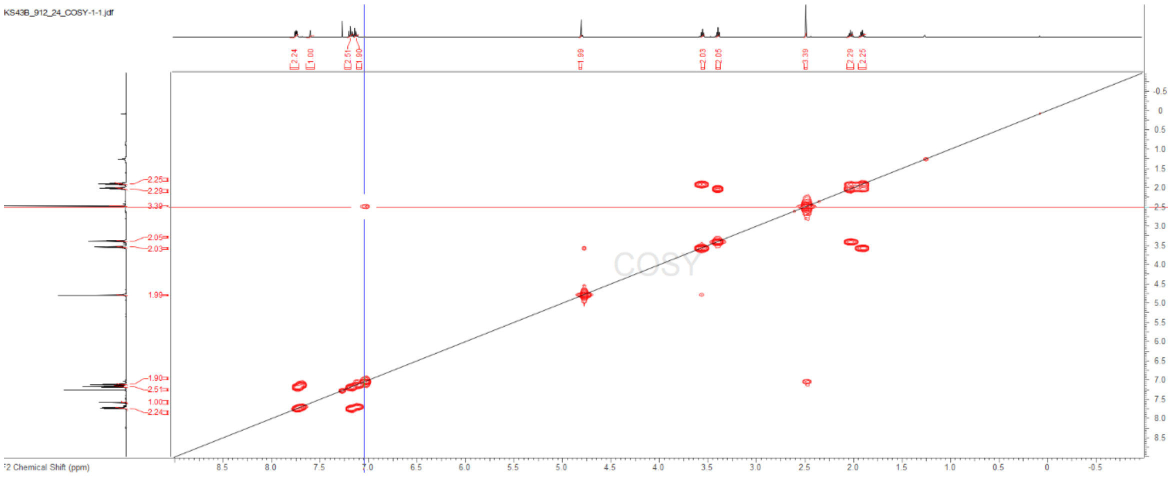

2-(2-(4-fluorophenyl)-5-methyl-1*H*-benzo[*d*]imidazol-1-yl)-1-(pyrrolidin-1-yl)ethan-1-one (10)

LC-MS (ESI) calcd for $C_{20}H_{20}FN_3O$: 338.16 [M + H$^+$], found: 338.232 [M + H$^+$].

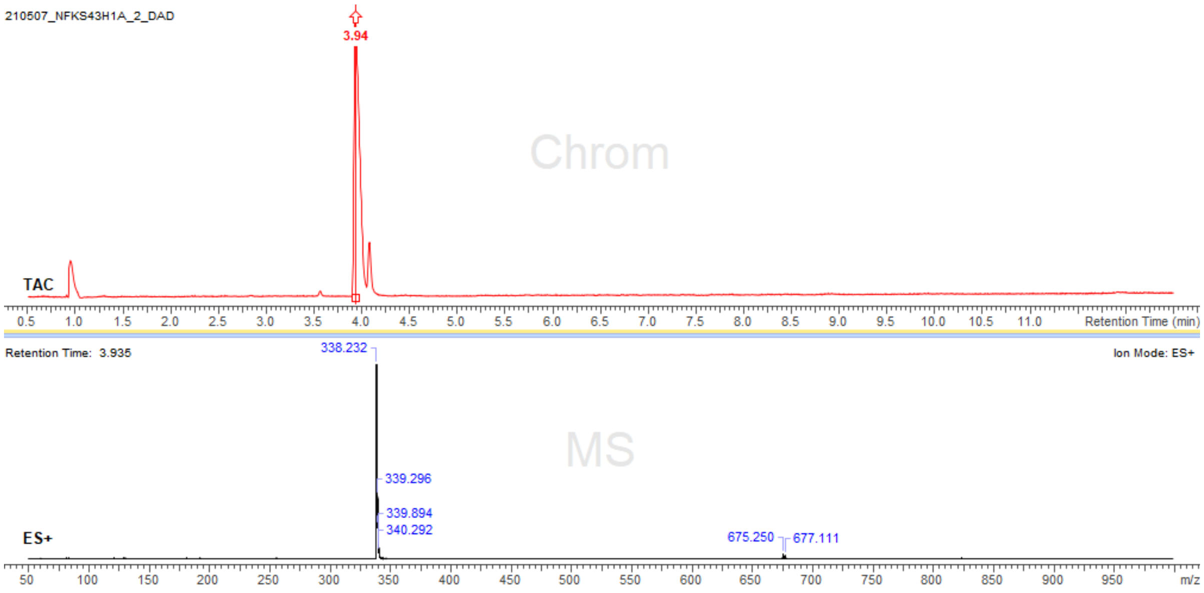

¹HNMR (500 MHz, $CDCl_3$)

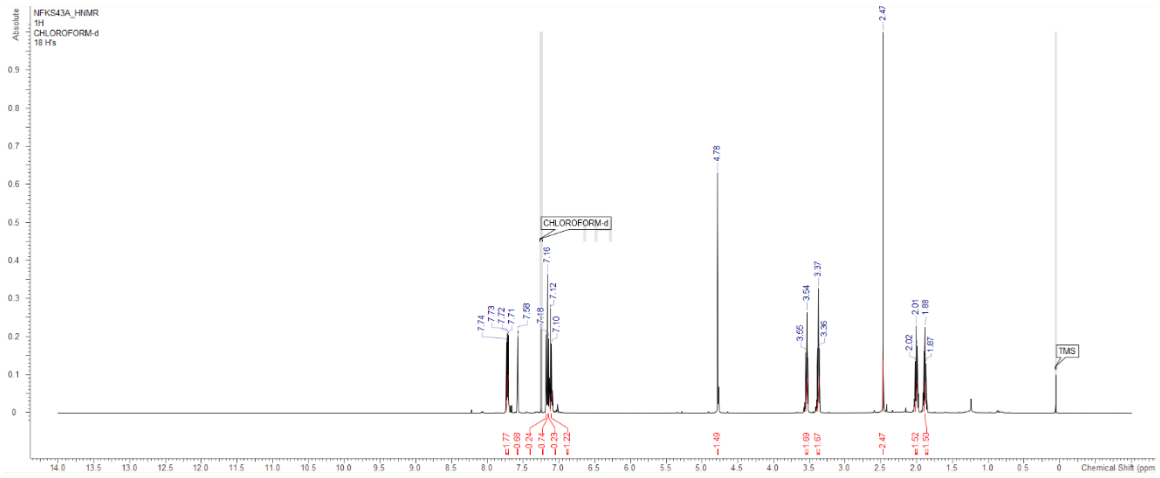

^{13}C NMR (126 MHz, $CDCl_3$)

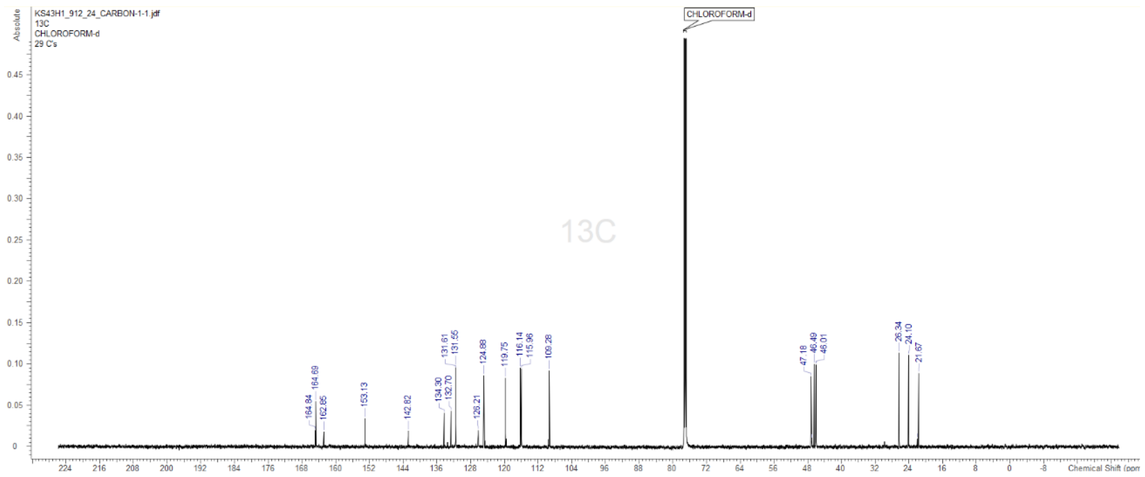

COSY (500 MHz, $CDCl_3$)

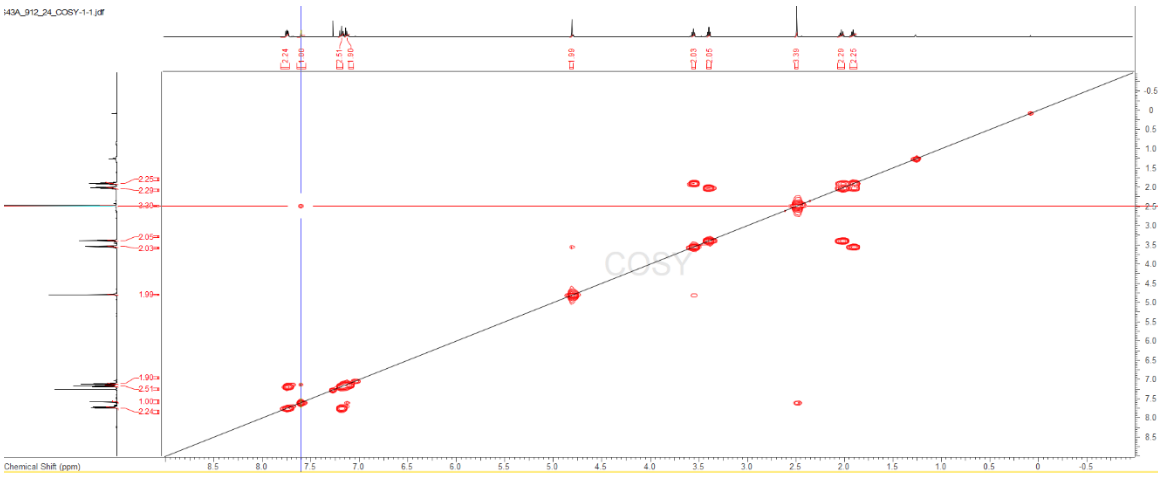

2. LCMS/MS chromatograms of the tested compounds in the metabolic stability assay

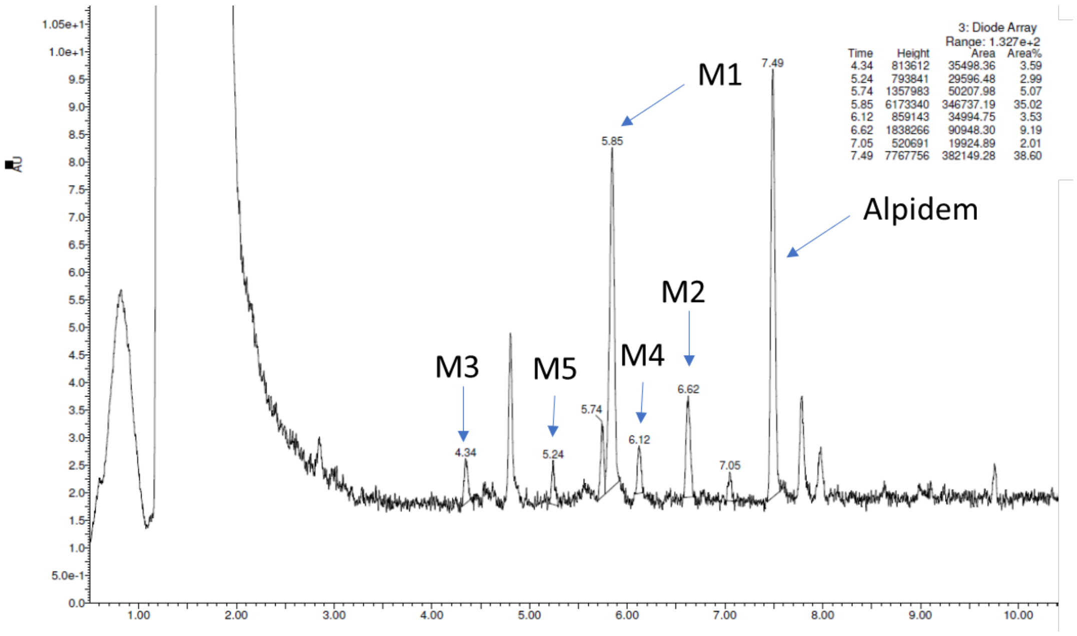

Figure 1. UPLC of the reaction mixture after 120 min incubation of compound Alpidem with HLMs.

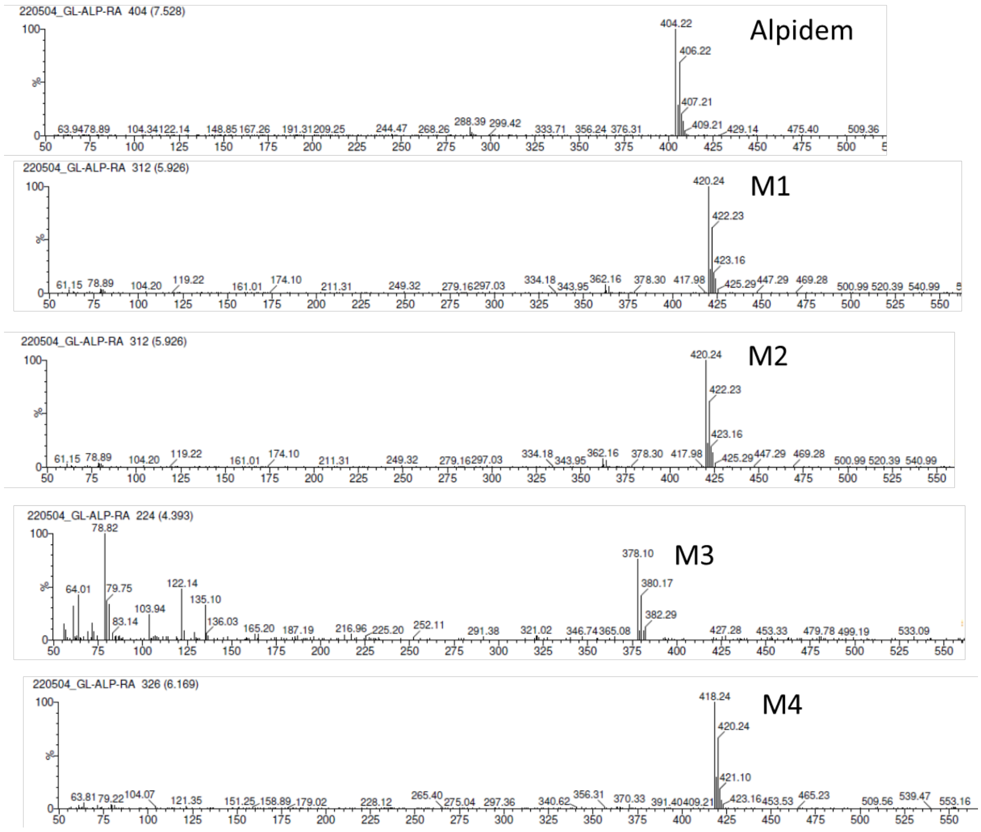

Figure 2. The mass spectra of Alpidem and its metabolites M1-M4 obtained after incubation with HLMs.

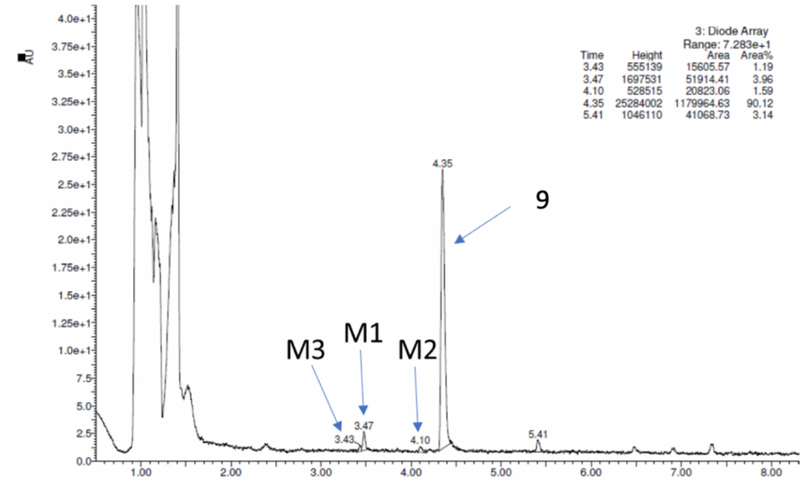

Figure 3. UPLC of the reaction mixture after 120 min incubation of compound **9** with HLMs.

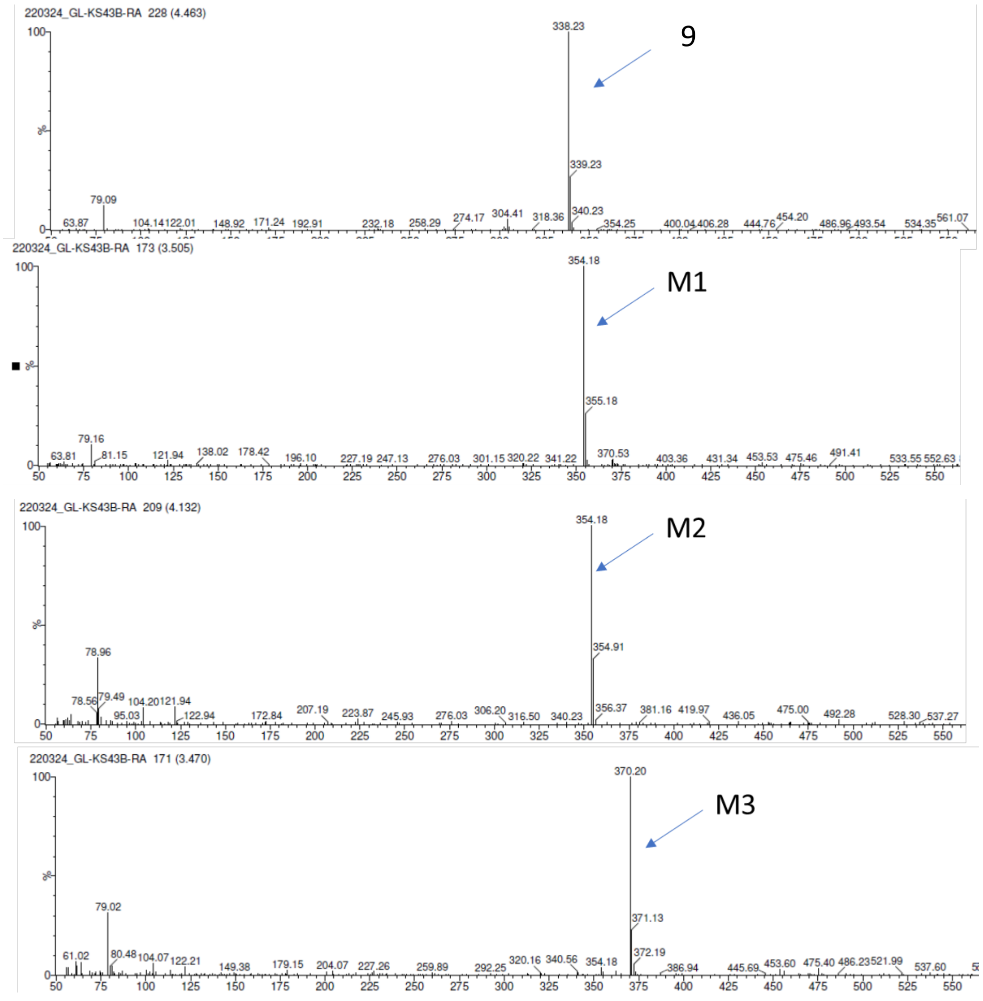

Figure 4. The mass spectra of **9** and its metabolite M1 obtained after incubation with HLMs

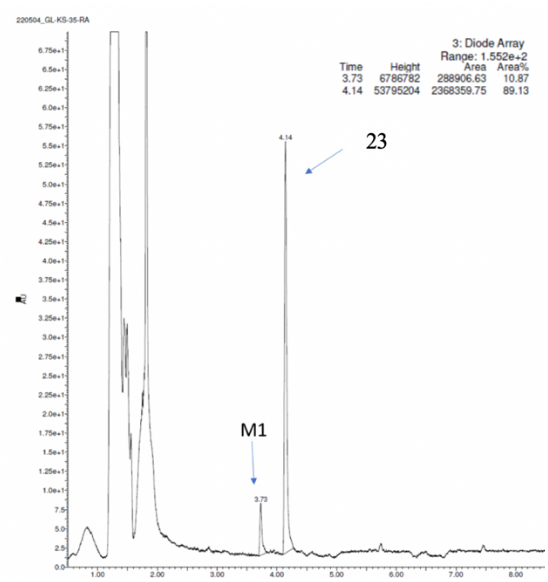

Figure 6. UPLC of the reaction mixture after 120 min incubation of compound **23** with HLMs.

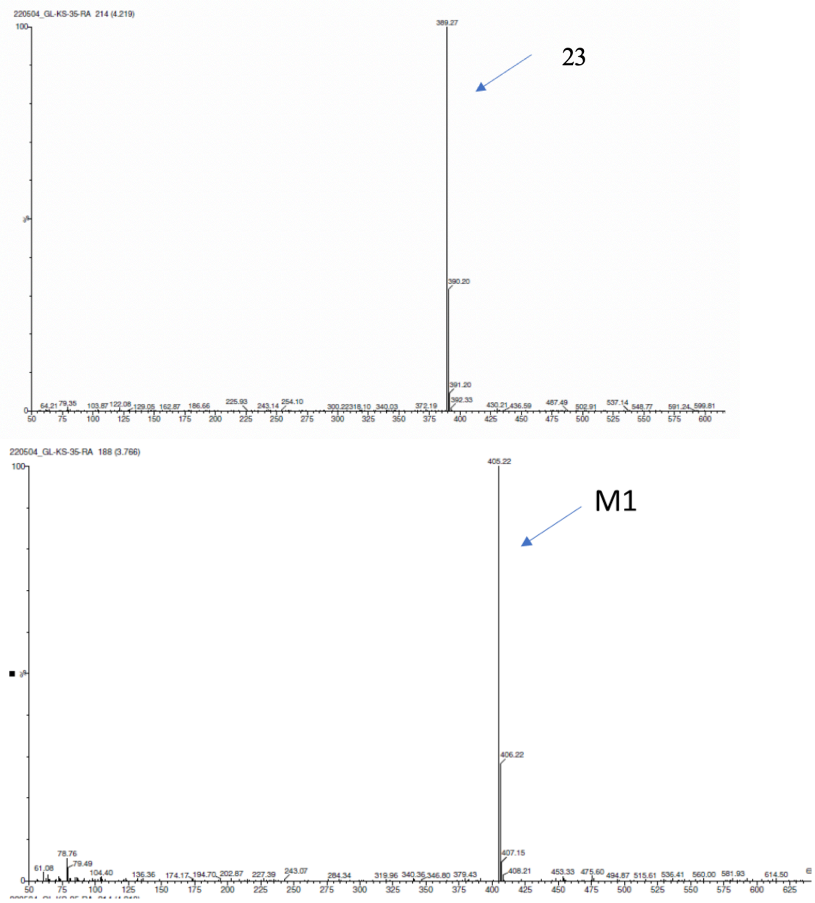

Figure 4. The mass spectra of and its metabolite M1 obtained after incubation with HLMs.
